# Context-Specific Efficacy of Apalutamide Therapy in Preclinical Models of *Pten*-Deficient Prostate Cancer

**DOI:** 10.3390/cancers13163975

**Published:** 2021-08-06

**Authors:** Marco A. De Velasco, Yurie Kura, Naomi Ando, Noriko Sako, Eri Banno, Kazutoshi Fujita, Masahiro Nozawa, Kazuhiro Yoshimura, Kazuko Sakai, Kazuhiro Yoshikawa, Kazuto Nishio, Hirotsugu Uemura

**Affiliations:** 1 Department of Urology Kindai, University Faculty of Medicine, Osaka-Sayama 589-8511, Japan; y.kurakindai@med.kindai.ac.jp (Y.K.); ulo_norika@yahoo.co.jp (N.A.); miminoki216@gmail.com (N.S.); ebanno@med.kindai.ac.jp (E.B.); fujita@uro.med.osaka-u.ac.jp (K.F.); nozawa06@med.kindai.ac.jp (M.N.); yoshimur@med.kindai.ac.jp (K.Y.); 2 Department of Genome Biology, Kindai University Faculty of Medicine, Osaka-Sayama 589-8511, Japan; kasakai@med.kindai.ac.jp (K.S.); knishio@med.kindai.ac.jp (K.N.); 3 Research Creation Support Center, Aichi Medical University, Nagakute 480-1195, Japan; yoshikaw@aichi-med-u.ac.jp

**Keywords:** prostate cancer, androgen receptor, preclinical efficacy, apalutamide, castration resistance, PTEN

## Abstract

**Simple Summary:**

Next-generation antiandrogens have transformed the therapeutic landscape for castration-resistant prostate cancer. Their utility in other indications, such as high-risk castration-sensitive cancers and as combination therapy, are being investigated. Our aim was to profile the in vivo antitumor activity of apalutamide in phenotypically distinct mouse models of *Pten*-deficient castration-naïve and castration-resistant prostate cancer, using both early- and late-stage disease models, and to profile the molecular responses. We also evaluated the therapeutic potential and characterized the molecular responses of the combined targeted AR/AKT blockade and showed that while this approach was promising in vitro, it was mostly ineffective in vivo, particularly in the castration-resistant setting. Our findings provide evidence that links therapeutic resistance to STAT3 and PIM-1 in the castration-resistant setting and provide insights into the context-specific antitumor activity of apalutamide.

**Abstract:**

Significant improvements with apalutamide, a nonsteroidal antiandrogen used to treat patients suffering from advanced prostate cancer (PCa), have prompted evaluation for additional indications and therapeutic development with other agents; however, persistent androgen receptor (AR) signaling remains problematic. We used autochthonous mouse models of *Pten*-deficient PCa to examine the context-specific antitumor activity of apalutamide and profile its molecular responses. Overall, apalutamide showed potent antitumor activity in both early-stage and late-stage models of castration-naïve prostate cancer (CNPC). Molecular profiling by Western blot and immunohistochemistry associated persistent surviving cancer cells with upregulated AKT signaling. While apalutamide was ineffective in an early-stage model of castration-resistant prostate cancer (CRPC), it tended to prolong survival in late-stage CRPC. Molecular features associated with surviving cancer cells in CRPC included upregulated aberrant-AR, and phosphorylated S6 and proline-rich Akt substrate of 40 kDa (PRAS40). Strong synergy was observed with the pan-AKT inhibitor GSK690693 and apalutamide in vitro against the CNPC- and CRPC-derived cell lines and tended to improve the antitumor responses in CNPC but not CRPC in vivo. Upregulation of signal transducer and activator of transcription 3 (STAT3) and proviral insertion in murine-1 (PIM-1) were associated with combined apalutamide/GSK690693. Our findings show that apalutamide can attenuate *Pten*-deficient PCa in a context-specific manner and provides data that can be used to further study and, possibly, develop additional combinations with apalutamide.

## 1. Introduction

For eight decades, the androgen receptor (AR) has been the primary target for advanced prostate cancer and is thus one of the most studied proteins. Targeting prostate cancer with hormonal treatment is initially effective; however, the treatment eventually fails, giving rise to castration-resistant prostate cancer (CRPC). Approaches targeting AR signaling have evolved and newer methods aimed at inhibiting AR function by impeding its transcriptional activity have been approved for clinical use. Despite significant improvements in clinical outcomes, prostate cancer in patients receiving next-generation AR therapies either fails to respond or ultimately develops resistance to the treatments. Mechanisms for acquired resistance to next-generation antiandrogens, such as enzalutamide, apalutamide and abiraterone acetate plus prednisolone/prednisone, have been attributed to AR alterations such as mutations in the ligand-binding domain or the presence of truncated AR splice variants [1,2]. Another mechanism by which AR signal activation continues in CRPC is crosstalk with pro-survival pathways [3].

Prostate cancers are characterized by a high degree of inter- and intra-tumor heterogeneity that results from a combination of host genetic and epigenetic factors, influence of tumor microenvironments and adaptive responses anticancer therapies. Molecular mechanisms that drive cancer progression are founded on the complex molecular interactions between diverse cell populations enabling tumors to reprogram intracellular regulatory networks, allowing to them further progress and survive [4]. The PI3K/AKT signal pathway is a master regulatory pathway that is essential for modulating vital biological processes and preserving normal cell homeostasis; however, it is frequently altered in human prostate cancer and is implicated in castration resistance [5,6]. Aberrant PI3K/AKT signaling results primarily through the loss of the phosphatase and tensin homolog (PTEN), which occurs from deletion or mutations but can also be attributed to *PIK3CA/B* mutations as well as other AKT-activating mutations [7]. Loss of the tumor suppressor PTEN frequently occurs from deletion or mutation and is associated with increased prostate cancer progression, AR-independent growth and poor patient prognosis [8]. Additional reports also show that loss of PTEN function can facilitate the progression to androgen independent prostate cancer by enabling AR activation [9]. Preclinical models also provide evidence to implicate PTEN deficiency and high levels of AR splice variants, which are also implicated in castration resistance [10,11,12]. Conversely, decreased AR expression and AR-responsive gene signaling is often observed in *PTEN*-deficient prostate cancer cells and, in this setting, inhibition of PI3K/AKT signaling results in the reactivation of AR through a reciprocal feedback loop between AR and AKT [13,14,15].

Apalutamide is a non-steroidal antiandrogen that binds directly to the ligand-binding domain of AR, preventing its nuclear translocation and binding to DNA, thus impeding AR-mediated transcription similarly to that of enzalutamide, but with greater efficacy, as shown in murine models [16]. ERLEADA^®^ (apalutamide) received its first U.S. Food and Drug Administration approval in 2018 for treating men with non-metastatic CRPC and was expanded to men with metastatic castration-sensitive prostate cancer (mCSPC) in 2019. Apalutamide continues to be evaluated for additional indications and its efficacy with other treatments, including other antiandrogens, radiotherapy, chemotherapy, and immunotherapy, is being assessed. Yet, there are few reports that explore and detail its basic intricacies in a context-specific setting of prostate cancer, or how it interacts with other therapeutic agents.

In this study, we characterized the antitumor activity of apalutamide in well-established, genetically engineered mouse models of *Pten*-deficient prostate cancer, modeled to reflect castration-naïve prostate cancer (CNPC) and CRPC in early cancer and late-stage disease settings. We also evaluated the efficacy of a combination therapy of apalutamide and an AKT inhibitor and identified the potential targets associated with therapeutic resistance.

## 2. Results

### 2.1. Apalutamide Shows Antitumor Efficacy in Mouse Castration-Naïve Pten-Deficient Prostate Cancer

**Figure 1:**
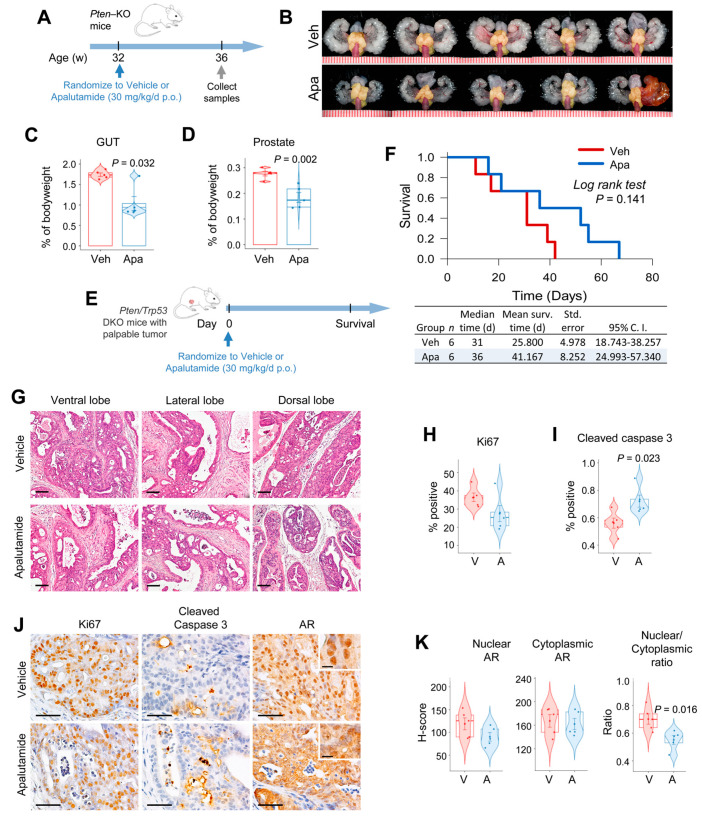
Apalutamide inhibits *Pten*-deficient castration-naïve prostate cancer (CNPC) growth. (**A**) The antitumor efficacy of apalutamide was evaluated in an early-stage prostate cancer intervention model using *PSA^Cre^; Pten^loxP/loxP^* (KO) mice (*n* = 5 mice/group). (**B**) Representative images of the genitourinary tracts (GUTs) collected en bloc; prostate tumors are highlighted in yellow. Scale represents mm. The effect of apalutamide on normal secondary sex organs and prostate tumors was evaluated by comparing the genitourinary tract (GUT) weights (**C**) and antitumor activity was assessed by comparing the weights of the dissected prostates (**D**). (**E**) The therapeutic benefit of apalutamide was assessed in a late-stage model of advanced prostate cancer using *PSA^Cre^; Pten^loxP/loxP^/Trp53^loxP/loxP^* (DKO) mice randomized to treatment when the palpable tumors reached 5 mm. (**F**) Kaplan–Meier plots showing overall survival curves for mice after the indicated treatment. (**G**) Representative H&E-stained sections of KO mouse prostate tumors from (**A**) are shown. Scale bars = 250 μm. Immunohistochemical (IHC) quantification of cancer cell proliferation (**H**) and apoptotic rates (**I**) in KO mice treated with apalutamide. (**J**) Representative photomicrographs of prostate tumors from (**A**) immunostained with the indicated antibodies. Scale bars = 100 μm and the inserted scale bar = 10 μm. (**K**) IHC quantification of AR expression in mice from (**A**). For figure plots, horizontal bars represent the mean ± SE; center lines show the medians and box limits indicate the 25th and 75th percentiles; the violin plots show the distribution and the circles represent individual samples. Unless indicated, *p* values indicate significance of the Student *t*-test.

We assessed the antitumor activity of apalutamide using two well-established mouse models of *Pten*-deficient prostate cancer [17,18]. In the *Pten*-KO intervention model, 32-week-old tumor-bearing mice received four weeks of treatment with apalutamide (30 mg/kg, p.o. 5 times/week p.o., *n* = 5) or the vehicle (CMC p.o., *n* = 5, Figure 1A). Apalutamide-treated mice had significantly reduced GUT weights (Figure 1B,C). Since the GUT consists of both normal genitourinary organs as well as prostate tumors, the decrease in size indicates atrophy of both normal and tumor tissues related to apalutamide treatment. However, in this model, prostate weight, which includes tumors, is a better surrogate for tumor burden, and the apalutamide-treated mice demonstrated a significant reduction in prostate weight (33.5%) compared to the vehicle-treated controls (Figure 1B–D). Since both normal accessory sex organs and prostate tumors express, and are regulated by, AR, a reduction in size after treatment with apalutamide shows that it is active in this model.

We next evaluated the therapeutic impact of apalutamide therapy by examining its effect on survival in conditional *Pten/Trp53* double knockout (DKO) mice employing a model of advanced castration-naïve prostate cancer (Figure 1E and Supplementary Figure S1A). In this setting, treatment with apalutamide (30 mg/kg, p.o. 5 times/week p.o., *n* = 6) tended to prolong the cumulative survival times over the vehicle controls (*n* = 6), with the median survival being 36 versus 31 days (*p* = 0.141), respectively (Figure 1E,F). Moreover, although statistically insignificant, the apalutamide-treated mice experienced a 1.7-fold increase in time to progression and post-progression survival (Supplementary Figure S1B,C). Since mice in this model are euthanized when tumors reach the maximum target sized, the final tumor burden is not a valid indicator of antitumor activity, rather, larger tumor volumes are usually associated with longer survival times [18]. In this study, final tumor burden was indeed greater in the apalutamide-treated mice; however, using the final tumor burden and survival time, we inferred the relative tumor growth rates, and using this surrogate we observed that the relative tumor growth rate was higher in the vehicle control group (Supplementary Figure S1D).

We next examined the histopathological changes associated with apalutamide therapy in prostate tumors from *Pten*-KO mice in Figure 1A. Histological changes associated with androgen deprivation, such as atrophy, decrease in the size of malignant glands, loss of luminal space and increased stroma, were observed in prostate tumors from mice treated with apalutamide. Notably, these histological changes were present in all lobes of the mouse prostate, but the effects were more prominently seen in the ventral and dorsal lobes (Figure 1G). Apalutamide-treated mice also had a 25% lower cancer cell proliferation rate versus the vehicle control, as determined by Ki67 IHC (Figure 1H,J). Treatment with apalutamide also significantly increased the apoptotic rate two-fold, as measured by cleaved caspase-3 IHC. To confirm the molecular activity of apalutamide in our model, we examined AR expression by IHC. Overall, the extent of cytoplasmic AR did not vary between the vehicle and apalutamide-treated mice; however, mice treated with apalutamide showed decreases in the percentage and intensity of nuclear staining as well as the AR nuclear/cytoplasmic ratio, although the latter will need to be confirmed using additional techniques, such as cell fractionation (Figure 1J,K). These results show that apalutamide has antitumor activity in mouse *Pten*-deficient CNPC.

### 2.2. Molecular Characterization of Apalutamide in Pten-Deficient CNPC

**Figure 2:**
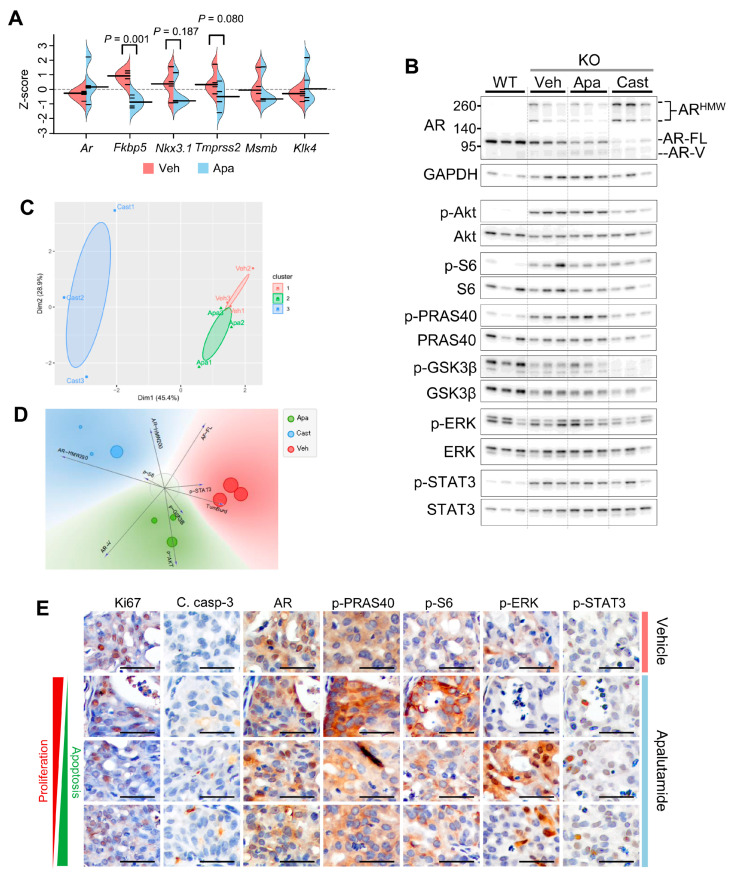
Profiling *Pten*-deficient castration-naïve prostate cancer (CNPC) response to apalutamide therapy. Prostates from a subset of mice (*n* = 4 mice/group) in Figure 1A were utilized to profile the effects of apalutamide therapy on androgen receptor (AR) transcriptional activity. (**A**) Analysis of *Ar* and AR target genes by qRT-PCR: violin plots show the distribution; long beanlines indicate the mean and short beanlines represent individual samples; *p* values indicate significance of the Student *t*-test. The effects on the molecules associated with survival pathways were compared between four weeks of treatment with the vehicle, apalutamide (30 mg/kg, p.o. 5 times/week) and surgical castration in *Pten*-deficient (CNPC). Samples were collected four hours after the last treatment dose and a normal prostate from wildtype (WT) mice were included for reference. Samples were analyzed by semi quantitative Western blot analysis with ImageJ. (**B**) Western blots of full-length AR (AR-FL), AR splicing variant (AR-V), AR high-molecular weight (AR-HMW) and the signaling molecules associated with PI3K/AKT, MAPK and STAT3 signaling. Semiquantitative densitometric analysis was performed in ImageJ; proteins were first normalized to GAPDH and phosphorylated proteins were normalized to total protein. (**C**) Supervised clustering by partitioning around medoids of semiquantitative Western blot data illustrates distinct delineation between treatment responses in tumor samples. (**D**) Freeviz plot showing visual multivariate correlates to treatment. Length of the vectors indicate strength of association and marker size corresponds to tumor burden. (**E**) Photomicrographs of matched areas of immunostained serial sections of CNPC treated with vehicle or apalutamide show the association between cancer cell proliferation (Ki67) and apoptosis (cleaved caspase-3) and markers related to cancer survival signaling pathways. Scale bars = 50 μm. See Supplementary Figure S2E for a low magnification version.

We next performed characterization studies to confirm the molecular activity of apalutamide and gain further insight into its interactions with signaling pathways frequently altered in PTEN-deficient prostate cancer. Prostates from *Pten*-KO mice treated with apalutamide (30 mg/kg, p.o. 5 times/week p.o., *n* = 4) or the vehicle (CMC p.o., *n* = 4) for four weeks were used to measure the mRNA expression levels of a set of AR target genes. The mRNA levels of the AR target genes *Fkbp5*, *Nkx3*.1, *Tmprss2* and *Msmb* were lower in tumors from mice treated with apalutamide (Figure 2A). Prostate cancers can adapt to androgen deprivation by activating alternate signaling pathways in both AR-dependent and -independent manners; thus, we next aimed to examine the effect of apalutamide on AR protein expression and that of marker molecules for Akt, mitogen-activated protein kinase (MAPK) and of signal transducer and activator of transcription 3 (STAT3_ signaling [19]. For this, we first examined the protein expression levels of AR as well as phosphorylated and total protein levels of AKT^pS473^, S6, PRAS40, glycogen synthase kinase-3 beta (GSK3β), ERK and STAT3^pY705^ in prostate tumors from *Pten*-KO mice after receiving four weeks of treatment with vehicle or apalutamide. We also compared the effects of apalutamide to that of surgical castration (post four weeks) and we used normal prostate from wildtype mice as a reference (Figure 2B). Semiquantitative analysis of the Western blot densitometry showed that expression levels of full-length AR (AR-FL) were lower in prostate tumors compared to normal prostate (Supplementary Figure S2A). However, compared to normal prostate, the aberrant AR form, including those of an AR splice variant (AR-V) with a molecular weight of ~80 kD and those with a molecular weight ~220 and ~250 kD (AR-HMW^220^ and AR-HMW^250^, respectively), were prominently expressed and elevated in tumor samples (Figure 2B, and Supplementary Figure S2A). These aberrant forms of AR have been detailed in our cancer model and are specific for AR; however, their functional relevance has not been determined [12]. Notably, levels were highest in tumors from mice receiving apalutamide and the AR-HMW200/250 levels were highest in tumors from surgically castrated mice (Figure 2B, and Supplementary Figure S2A).

As hypothesized, *Pten*-deficient tumors expressed higher levels of phosphorylated (p)-AKT^pS473^ and its downstream molecules p-S6 and p-PRAS40 (Figure 2B, and Supplementary Figure S2B) compared to the normal prostate. Overall expression levels of p-ERK did not differ between the normal prostate and tumors, but levels of p-STAT^pY705^ were greater in the tumors. We next examined the effects of apalutamide and androgen deprivation treatments on these molecules in *Pten*-deficient tumors and the association between their expression levels and tumor burden. We used unsupervised and supervised clustering approaches to assess the similarities based on their normalized protein expression patterns. As a result, distinct clustering was observed between treatments (Figure 2C and Supplementary Figure S2C). Additionally, we used a multivariate visualization approach to identify p-AKT, AR-V and p-GSK3β as factors associated with apalutamide treatment (Figure 2D) and used Pearson correlation to identify positive correlations between tumor burden, AR-V, p-AKT^pS473^, P-S6, p-PRAs40 and p-STAT^pY705^ in the apalutamide-treated tumors and tumor burden, AR-HMW^250^ and p-STAT^pY705^ in surgically castrated mice (Supplementary Figure S2D).

Tumor regions from the apalutamide-treated mice with sustained cancer cell proliferation and/or low apoptotic rates likely represent instances of poor response or inherent resistance. Thus, we aimed to explore this notion by characterizing the active tumor regions in apalutamide-treated mice with IHC to identify or associate features associated with cancer cell survival. Examination of serial tissue sections of prostate tumors from control and apalutamide-treated mice were stained with proliferation and apoptosis markers as well as AR, p-AKT^pS473^, p-PRAS40, p-ERK and pSTAT3^pY705^ (Figure 2E and Supplementary Figure S2E). In apalutamide-treated mice, areas characterized with higher proliferation rates, decreased apoptosis and nuclear AR also expressed higher levels of p-PRAS40 and p-S6 (Figure 2E and Supplementary Figure S2E). It was also interesting to note that tumor regions with high p-ERK expression tended to be associated with a greater number of cleaved caspase-3-positive cells in apalutamide-treated mice.

### 2.3. Antitumor Activity of Apalutamide Monotherapy in Pten-Deficient CRPC

**Figure 3:**
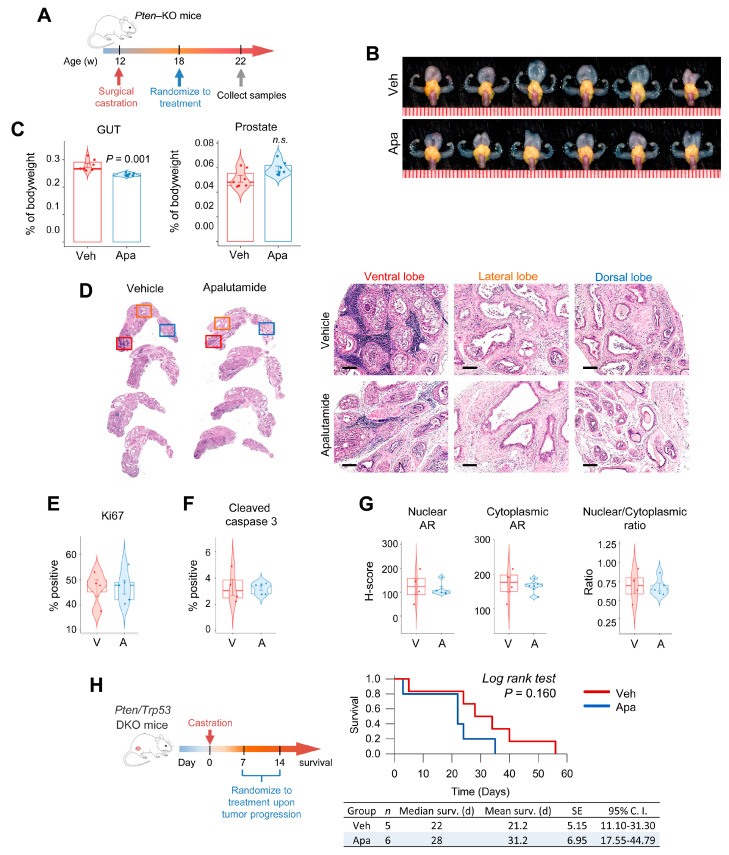
Antitumor activity of apalutamide in mouse *Pten*-deficient castration-resistant prostate cancer (CRPC). (**A**) Apalutamide (Apa, 30 mg/kg, p.o. 5 times/week) or the vehicle (Veh) were administered to *PSA^Cre^; Pten^loxP/loxP^* (KO) mice (*n* = 6 mice/group) evaluated in an early-stage model of CRPC. (**B**) Representative images of the genitourinary tracts (GUTs) collected en bloc; prostate tumors are highlighted in yellow. Scale represents mm. (**C**) The effect of apalutamide on normal secondary sex organs and prostate tumors was evaluated by comparing the genitourinary tract (GUT) weights, and antitumor activity was assessed by comparing the weights of the dissected prostates. Horizontal bars represent the mean ± SE, center lines show the medians and box limits indicate the 25th and 75th percentiles, and the violin plots show the distribution. (**D**) Representative H&E-stained sections of KO mouse prostate tumors are shown as low magnification scans with corresponding high magnification photomicrographs. Scale bars = 250 μm. Immunohistochemical (IHC) quantification of cancer cell proliferation (**E**), apoptotic rates (**F**) and AR expression (**G**) in KO mice treated with the vehicle (V) or apalutamide (A). Horizontal bars represent the mean ± SE, center lines show the medians and box limits indicate the 25th and 75th percentiles, and the violin plots show the distribution with the circles representing individual samples. (**H**) The therapeutic benefit of apalutamide was assessed in a late-stage model of advanced CRPC using *PSA^Cre^; Pten^loxP/loxP^/Trp53^loxP/loxP^* (DKO), as illustrated, and the Kaplan–Meier plots compare the overall survival curves for mice according to treatment. Unless indicated, *p* values indicate significance of the Student *t*-test.

We next examined the activity of apalutamide in the context of CRPC. For this we used the mouse CRPC intervention model in which the mice were orchidectomized six weeks before starting treatments with the vehicle (*n* = 7) or apalutamide (30 mg/kg, p.o. 5 times/week, *n* = 7) for a period of four weeks (Figure 3A). While the overall genitourinary tract (GUT) weight was lower in the apalutamide-treated mice, prostates weights tended to be greater (Figure 3B,C). In this model, androgen deprivation leads to atrophy of normal glands while castration-sensitive glands appear as dilated and cystic, and castration-resistant regions are characterized with the presence of regions of high-grade prostatic intraepithelial neoplasia (HG-PIN), which are more prominent in the ventral and lateral lobes [12]. In this study, CRPC mice treated with apalutamide showed a greater frequency of atrophic glands and dilated cystic glands in all lobes but tended be more pronounced in the lateral lobe (Figure 3D). Despite a larger appearance, the tumor regions in the lateral and ventral lobes were characterized with a higher proportion of dilated tumor glands separated by broad regions of stroma. Overall inflammatory changes were observed in the vehicle and apalutamide-treated mice but were more prominent in the vehicle-treated tumors, particularly, granulocytic infiltration in the ventral lobes. Overall proliferation and apoptotic rates were similar between the groups (Figure 3E,F, Supplementary Figure S3A). No differences were detected between the overall AR expression patterns in the vehicle and apalutamide-treated tumors (Figure 3G); however, we did note patterns that correlated the regions of persistent nuclear AR to positive Ki67 and low cleaved caspase-3 in the apalutamide-treated mice (Supplementary Figure S3A).

We next tested apalutamide in a long-term survival model of advanced CRPC to determine its therapeutic benefit. Here, we saw that mice randomized to apalutamide after surgical castration had a 21% improvement in median overall survival time (Figure 3H). Cumulative survival times, which measure survival from the castration date, were similar between the castrated vehicle and castrated apalutamide-treated mice, but the mean survival times were improved by 19% (Supplementary Figure S3B,C) and the median post-progression survival was marginally better in apalutamide-treated mice (median time of 21 days compared to 15 days in control mice, *p* = 0.535, Supplementary Figure S3D). Lastly, using the final tumor burden and survival time, we inferred the relative tumor growth rates. Using this surrogate, the relative tumor growth rate for the apalutamide-treated mice was longer than that of the vehicle control (Supplementary Figure S3E).

### 2.4. Molecular Characterization of Apalutamide Pten-Deficient CRPC

**Figure 4:**
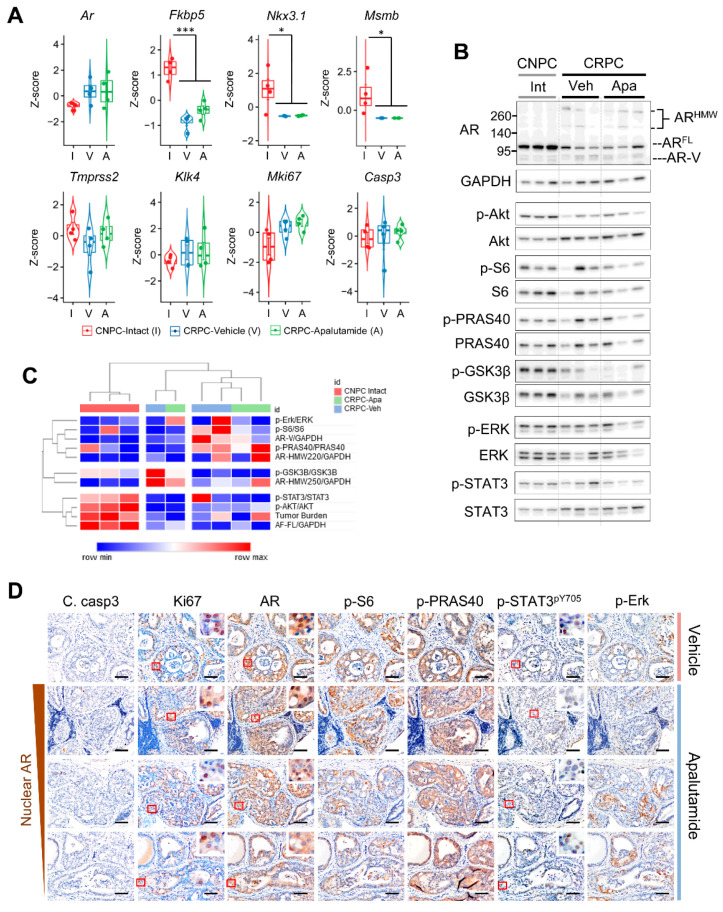
Profiling the molecular responses to apalutamide therapy in *Pten*-deficient castration-resistant prostate cancer (CRPC). Molecular responses to apalutamide therapy were determined by comparing the marker molecules of androgen receptor (AR) and prostate cancer cell survival signaling by qRT-PCR, Western blot and immunohistochemistry (IHC) in intact (Int) castration-naïve prostate cancer (CNPC) and CRPC after four weeks of treatment with the vehicle (Veh) or apalutamide (Apa, 30 mg/kg, p.o. 5 times/week). (**A**) qRT-PCR comparison of the mRNA of the *Ar* and AR target genes in intact CNPC (I) and CRPC treated with the vehicle (V) or apalutamide (A); *n* = 4 mice/group. Significance calculated by the Student–Newman–Keuls post-hoc test for individual comparisons, upon a significant one-way ANOVA (* *p* < 0.05 and *** *p* < 0.001). (**B**) Western blots of full-length AR (AR-FL), AR splicing variant (AR-V), AR high-molecular weight (AR-HMW) and the signaling molecules associated with PI3K/AKT, MAPK and STAT3 signaling in intact CNPC (Int) and CRPC treated with the vehicle (Veh) or apalutamide (Apa); *n* = 3 mice/group. Samples were collected four hours after the last treatment dose. Semiquantitative densitometric analysis was performed in ImageJ; the proteins were first normalized to GAPDH and phosphorylated proteins were normalized to total protein. (**C**) Heatmap and unsupervised hierarchical clustering of the z-score-transformed data of the semiquantitative Western blot data and tumor burden using average linkage and Euclidean distance. (**D**) Photomicrographs of the matched areas of immunostained serial sections of CRPC treated with the vehicle or apalutamide show the association between nuclear AR expression, cancer cell proliferation (Ki67), apoptosis (cleaved caspase-3) and the markers related to cancer survival signaling pathways. Scale bars = 50 μm.

We next examined the effects of apalutamide on the AR, PI3K/AKT, MAPAK and STAT3 signaling in CRPC. For this, we first examined the mRNA levels of AR and selected *Ar* target genes in a subset of mice from Figure 3A (*n* = 4 mice/group). Overall, the CRPC samples tended to have higher Ar expression than intact CNPC, and the levels were not affected by apalutamide treatment (Figure 4A). Conversely, the mRNA levels for *Fkbp5*, *Nkx3*.1 and *Msmb* were considerably diminished in all CRPC samples and, except for *Fkbp5*, which tended to be slightly higher in apalutamide treated mice, the levels did not vary much between the vehicle and apalutamide-treated samples (Figure 4A). We also examined the expression of *Mki67* and *Casp3*, which encode for Ki67 and Caspase-3, respectively. The mRNA levels of *Mki67* did not differ between the vehicle and apalutamide-treated mice; however, these levels tended to be higher in CRPC. Unlike CNPC, unsupervised clustering using mRNA expression levels of the subset of genes did not reveal a discernible difference between the vehicle and apalutamide tumors (Supplementary Figure S4A).

As before, we used Western blotting to profile the protein expression patterns of the AR and signaling molecules implicated in prostate cancer on a separate cohort of intact CNPC and vehicle and apalutamide-treated CRPC mice (*n* = 3 mice). Anti-tumor responses from this subset were consistent with our previous cohort (Supplementary Figure S4B). Analysis of semi-quantitative densitometry showed significantly reduced levels of AR-FL in CRPC compared to intact CNPC, although to a lesser degree in the apalutamide-treated mice (Figure 4B and Supplementary Figure S4C). Notably, aberrant AR (AR-V and high molecular weight AR) were generally higher in CRPC with AR-V and AR-HMW^220^ showing a higher tendency in the vehicle CRPC whereas AR-HMW^250^ tended to be higher in the apalutamide-treated tumors (Supplementary Figure S4C). While phosphorylated AKT^pS473^ levels were lower in both the vehicle and apalutamide-treated CRPC, no overt changes were observed with phosphorylation of the downstream targets, although p-S6 tended to be higher in vehicle CRPC and p-PRAS40 tended to higher in both the vehicle and apalutamide-treated CRPC (Supplementary Figure S4D). Overall, apalutamide did not appear to influence the levels of phosphorylated ERK; however, in this cohort, levels of p-STAT3^pY705^ tended to be lower in the apalutamide-treated CRPC (Supplementary Figure S4D). Unsupervised clustering showed distinct clusters between CNPC and CRPC but did not reveal associations or patterns with apalutamide treatment in CRPC (Figure 4C). Further characterization using Pearson correlation analyses did show positive correlations between tumor burden, p-AKT^pS473^, p-PRAS40 and aberrant AR (Supplementary Figure S4E). Multivariate visualization did not reveal any treatment-specific markers; however, aberrant AR seemed more likely to be associated with a high tumor burden in CRPC (Supplementary Figure S4F).

We took a closer examination of the serially immunostained sections of the active cancerous regions characterized by a lack apoptosis and persistent proliferation to provide us with additional details regarding resistance to apalutamide and CRPC cell survival. Primarily, persistent nuclear AR was associated with higher levels of p-S6 (Figure 4D). Interestingly p-PRAS40, another AKT substrate, was constitutively expressed in apalutamide-treated CRPC, regardless of nuclear AR status, and while p-STAT3 expression in cancer cells generally remained low in areas corresponding to nuclear AR, the proportion of strong-positive p-STAT3^py705^ tended to be greater in regions with low nuclear AR (Figure 4D). Lastly, p-ERK appeared to be present in regions characterized by moderate nuclear AR expression. Collectively, our results show that persistent AR activity is the likely mechanism of action for primary resistance to apalutamide in the *Pten*-deficient CRPC model. However, additional AR-independent mechanisms involving the STAT3 and MAPK pathways may also be implicated in apalutamide resistance.

### 2.5. Apalutamide and AKT Inhibition Synergize to Suppress Prostate Cancer Cell Growth In Vitro

**Figure 5:**
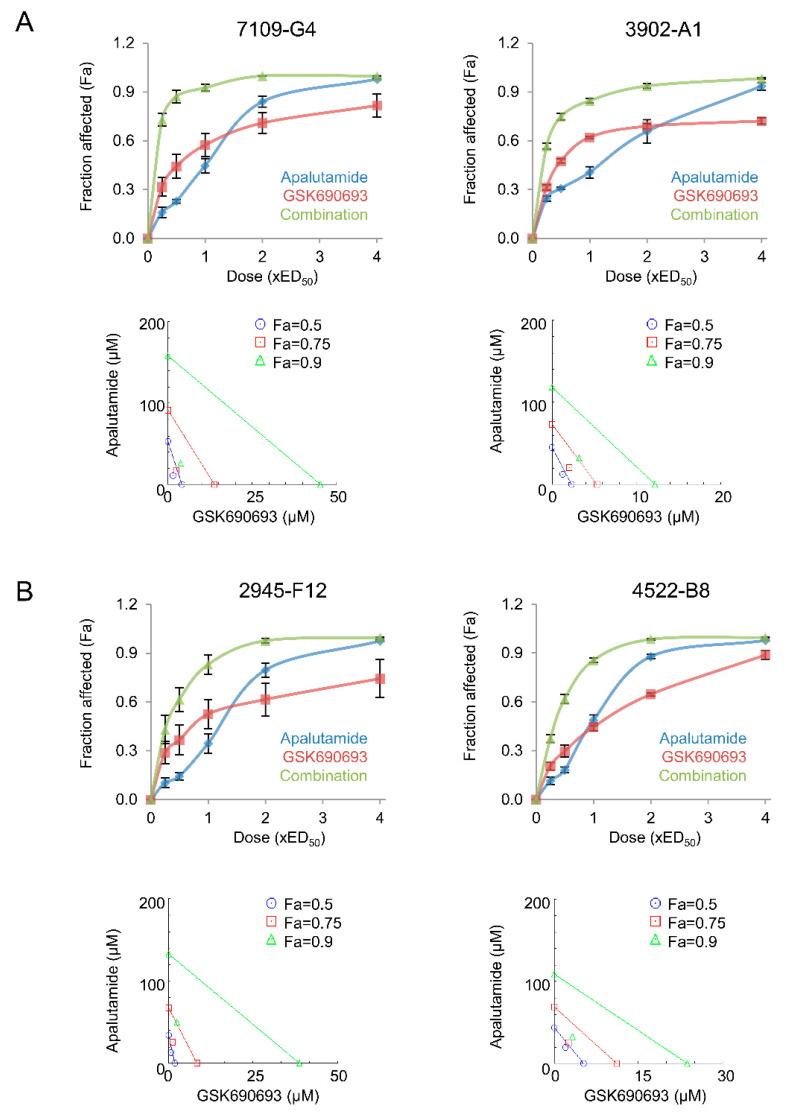
Apalutamide and AKT inhibition synergizes to suppress mouse prostate cancer cell viability in vitro. Mouse prostate cancer cell lines established from *Pten*-deficient castration-naïve prostate cancer, (CNPC) 7109-G4 and castration-resistant prostate cancer (CRPC), 2945-F12 and *Pten/Trp53*-deficient CNPC, 3902-A1 and CRPC, 4522-B8 were treated with apalutamide and GSK690693 alone or in combination for 96 h constant ratio combinations based on the effective doses resulting in 50% growth inhibition (ED_50_) and synergy measured by the Chou–Talalay combination index (CI), see Table 1. Dose–effect curves and representative isobolograms for CNPC- (**A**) and CRPC-derived (**B**) cells. Data represent the mean values ± SE of four independent experiments performed in quadruplicate.

Reciprocal feedback between AR and PI3K/AKT occurs in prostate cancer and is implicated in loss of therapeutic efficacy [13,14]. Moreover, we have shown that the antitumor effects of androgen withdrawal and Ar silencing could be enhanced with AKT inhibition [12,18]. Given the dependency of AR and PI3K pathway signaling in *Pten*-deficient prostate cancer, we next investigated if the antitumor activity of apalutamide could be enhanced with AKT inhibition. For this, we first examined the combinatorial effects of apalutamide and the pan-AKT inhibitor, GSK690693, in vitro using mouse prostate cancer cell lines derived from *Pten*-deficient CNPC (7109-G4) and CRPC (2945-F12), and *Pten/Trp53*-deficient CNPC (3902-A1) and CRPC (4522-B8). The mean effective dose for 50% growth inhibition (ED_50_) of apalutamide and GSK-690693 is shown in Table 1, as well as the combination index (CI) at ED_50_ for the mouse prostate cancer cell lines. Overall, all cell lines demonstrated strong synergy with the apalutamide/GSK690693 treatment combination (Figure 5A,B). The strongest synergy was observed in 7109-G4 and 2945-F12 (mean combination index (CI) at ED_50_ were 0.585 and 0.628, respectively; Table 1). 3902-A1 and 4522-B8 cells represent a more aggressive cancer type and had a mean CI at ED_50_ values of 0.730 and 0.791, respectively, which are still considered synergistic, though to a lesser degree than that of the *Pten^−/−^/Trp^+/+^* cancer cells.

### 2.6. Combinatorial Effects of Apalutamide and AKT Inhibition In Vivo

**Figure 6:**
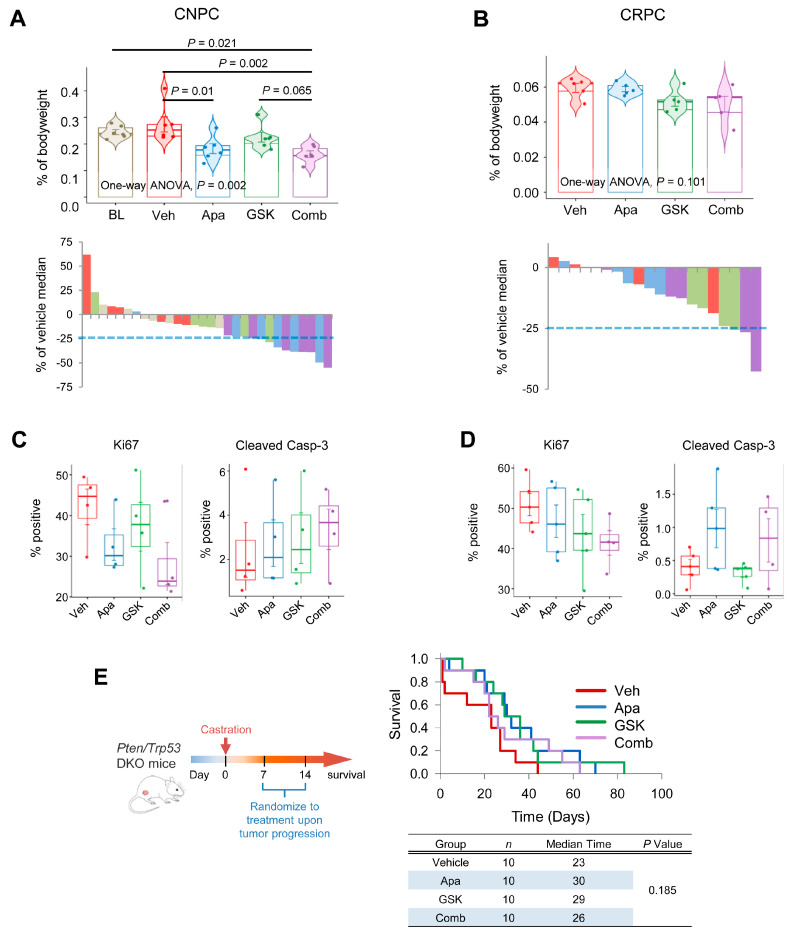
Efficacy evaluation of apalutamide and AKT inhibition in a mouse *Pten*-deficient prostate cancer. For castration-naïve prostate cancer (CNPC), 18-week-old *Pten*-KO mice were randomized to baseline (BL), vehicle control (Veh), apalutamide (30 mg/kg, p.o., 5 times/week), GSK690693 (GSK, 10 mg/kg, i.p., 5 times/week) or combination (Comb) groups (*n* = 6 mice/group) and were treated for four weeks. For castration-resistant prostate cancer (CRPC), 12-week-old *Pten*-KO mice were orchidectomized and randomized to Veh, Apa, GSK and Comb groups (*n* = 6 mice/group). Mice were dosed with the appropriate treatment four weeks beginning at 18 weeks of age. Comparison of tumor burden based on the prostate weight relative to bodyweight and waterfall plots showing the individual treatment responses in the CNPC (**A**) and CRPC (**B**) intervention models. Horizontal bars represent the mean ± SE, center lines show the medians and box limits indicate the 25th and 75th percentiles, and the violin plots show the distribution where circles represent the individual samples. *p* values indicate significance of the Student–Newman–Keuls post hoc test for individual comparisons, upon a significant one-way ANOVA. Immunohistochemical quantification of cancer cell proliferation (Ki67) apoptotic rates (cleaved caspase-3) in CNPC (**C**) and CRPC (**D**). Horizontal bars represent the mean ± SE, center lines show the medians and box limits indicate the 25th and 75th percentiles, and violin plots show the distribution where circles represent the individual samples; *n* = 4 samples/group. (**E**) The therapeutic benefit of apalutamide was assessed in a late-stage model of advanced CRPC using *PSA^Cre^; Pten^loxP/loxP^/Trp^53loxP/loxP^* (DKO), as shown, and the Kaplan–Meier plots compare the overall survival curves for mice according to treatment. Unless indicated, a *p* value indicates the significance of the *Log rank* test.

Given the synergistic activity of apalutamide and GSK690693 in vitro, we next evaluated the efficacy of this treatment combination in vivo. Using the *Pten*-KO model, we examined the effects of four weeks of treatment with apalutamide and GSK690693 as monotherapy and as combination therapy in CNPC and CRPC. In the CNPC model, we observed mean tumor burden reductions of 33.4%, 17.7% and 40.9% in apalutamide (30 mg/kg, p.o. 5 times/week.), GSK690693 (10 mg/kg, i.p. 5 times/week) and the combination regimens, respectively, versus the vehicle control (*n* = 6 mice/group, Figure 6A). Although the mean reduction in tumor burden was marginal, 5/6 (83.3%) of the mice treated with combination therapy achieved tumor reductions ≥25% compared to 3/6 (50%) for apalutamide or 1/6 (16.7%) for GSK690693. In the CRPC setting, tumor reductions of 1.0%, 12.8% and 15.5% were observed in mice treated with apalutamide (30 mg/kg, p.o. 5 times/week), GSK690693 (10 mg/kg, i.p. 5 times/week) and combination, respectively (*n* = 5 mice/group, Figure 6B). In this setting, 1/6 (16.7%) mice treated with GSK690693 and 2/6 (33.3%) of mice treated with combination reached tumor reductions ≥25% relative to the vehicle-treated mice. In CNPC, the cancer cell proliferation rates tended to be lower in the combination-treated mice compared to monotherapy, although these failed to reach statistical significance (Figure 6C). Additionally, the mean apoptotic rates tended to be higher with combination therapy in CNPC, whereas in CRPC, no difference was noted between the apoptotic rates of mice treated with apalutamide alone and those treated with apalutamide plus GSK690693 (Figure 6C,D).

We next sought to determine whether adding an AKT inhibitor to apalutamide would impact long-term survival in an advance model of CRPC. *Pten/Trp53*-DKO were randomized to the vehicle, apalutamide (30 mg/kg, p.o. 5 times/week), GSK690693 (10 mg/kg, i.p., 5 times/week) and apalutamide/GSK690693 combination therapy (*n* = 10 mice/group), using the model described in Figure 3H. In this model, overall survival times were improved with apalutamide or GSK690693 monotherapy, 30 and 29 days, respectively, versus 23 days in the vehicle controls (Figure 6E). However, combination therapy did not improve the median overall survival and was slightly lower than that of the monotherapy (median overall survival of 23 days). Together, these results indicate fundamental differences in sensitivities to combined AR/AKT inhibition with apalutamide and the pan-AKT inhibitor GSK690693, particularly in the context of *Pten*-deficient castration-sensitive and insensitive prostate cancer, as well as early-stage and late-stage cancer and/or *Trp53* status.

**Figure 7:**
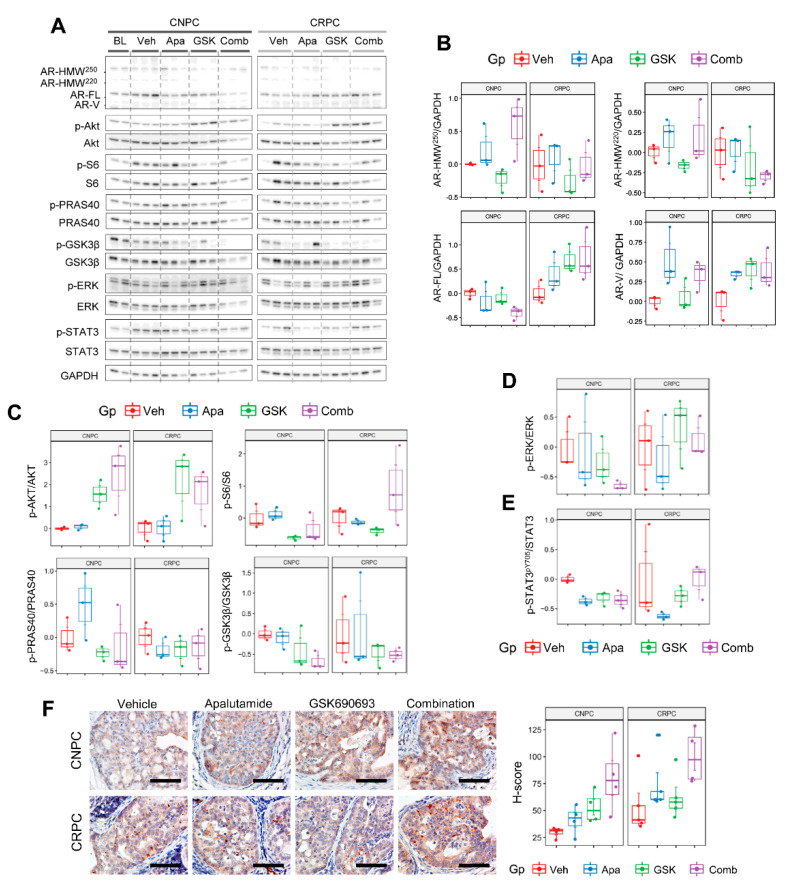
Molecular characterization of response to apalutamide/GSK690693 combination therapy in *Pten*-deficient prostate cancer. (**A**) A subset of *Pten*-deficient castration-naïve prostate cancer (CNPC) and castration-resistant prostate cancer (CRPC) samples from Figure 6A,B (*n* = 3 mice/group) were used to profile (**B**) full-length androgen receptor AR (AR-FL), aberrant AR (AR splicing variant (AR-V), AR high-molecular weight (AR-HMW)) and the signaling molecules associated with (**C**) PI3K/AKT, (**D**) MAPK and (**E**) STAT3 signaling by Western blot. Samples were collected four hours after the last treatment dose. Semiquantitative densitometric analysis was performed in ImageJ; proteins were first normalized to GAPDH and phosphorylated proteins were normalized to total protein. (**F**) Immunohistochemical visualization and quantification of PIM-1 expression in *Pten*-deficient prostate cancer after treatments with apalutamide and GSK690693 as monotherapy and combination therapy. Plots represent the PIM-1 H-scores in cancer cells as determined by QuPath. Horizontal bars represent the mean ± SE, center lines show the medians and box limits indicate the 25th and 75th percentiles, and the violin plots show the distribution where circles represent the individual samples. Scale bars = 100 μm.

We next profiled the protein levels in prostate tumors by Western blot using the same panel from the monotherapy experiments to confirm the molecular activity of GSK690693 and characterize the expression patterns in response to AR with apalutamide and AKT inhibition alone or as a combination therapy in a subset of mice from Figure 7A,B (Figure 7A). In this study set, an increase in the levels of AR-FL was seen primarily in treated CRPC (Figure 7B). Interestingly, elevated levels of aberrant AR appeared to be associated to apalutamide therapy in CNPC and CRPC, whereas AR-V expression levels were increased in apalutamide and apalutamide/GSK690693 in CRPC and in all pharmacologically treated mice in CRPC. Regarding marker molecules of the PI3K/AKT signaling pathway, the p-AKT^pS473^ levels were elevated following GSK690693 in both CNPC and CRPC; however, this phenomenon is often observed when using ATP-competitive inhibitors. Nevertheless, phosphorylation of the downstream substrates PRAS40 and GSK3β, and the mTOR downstream molecule S6 were suppressed in both models (Figure 7C). Furthermore, downregulation of these targets was improved or maintained with combination therapy in CNPC. In CRPC, however, repression of the AKT downstream molecules tended to subside with combination therapy and levels of S6 phosphorylation were notably increased. PI3K/AKT and AR signaling is also known to interact with MAPK and can promote cancer survival via compensatory activation [20]. In our study, levels of ERK phosphorylation were further diminished with combination therapy in CNPC and were largely unaffected in CRPC (Figure 7D). STAT3 is a transcription factor that is frequently overexpressed in CRPC and is associated with androgen-independent progression and resistance to enzalutamide [21]. We observed that in the CRPC cohort, the STAT3^pY705^ levels tended to be greater in mice treated with GSK690693 but not apalutamide; however, concomitant treatment with apalutamide and GSK690693 further increased the levels of STAT3 phosphorylation (Figure 7E). Given the increased activity of STAT3 in CRPC and lack of potent inhibition of the AKT signaling molecules, we examined the expression of PIM-1 (Proviral integration site for Moloney murine leukemia virus-1) in our model, particularly since it is a STAT3 target that is also associated with mediating resistance to AKT inhibitors [22]. IHC profiling showed that PIM-1 was primarily localized in cancer cells and tended to be weakly expressed in untreated CNPC, whereas it was moderately but constitutively expressed in CRPC and apalutamide-treated CNPC (Figure 7F). Additionally, PIM-1 expression tended to be greater in mice treated with apalutamide in both CNPC and CRPC, with substantial increases in combination-treated cohorts. Together, these results shed light into the context-specific responses of apalutamide when used as combination therapy with AKT inhibition in PTEN-deficient prostate cancer and provide additional evidence to further explore additional combination approaches to improve apalutamide therapeutic responses.

## 3. Discussion

Despite significantly improved outcomes for patients from next generation antiandrogen therapies, advanced prostate cancer eventually progresses and results in patients succumbing to the disease, which underscores the unmet need to continue to develop improved treatment strategies. Moreover, prostate cancer follows a well-defined series of states that are based on various clinical features associated with its progression, which range from clinically localized disease to metastatic CRPC. Given this dynamic range, treatment responses will certainly vary with each setting and will most likely be context specific. Cellular heterogeneity is a hallmark of solid cancers and one that is difficult to recreate in conventional cell-based preclinical models. To better assess the efficacy of the therapeutic agents requires the use of preclinical models that closely resemble the human disease. Our models are based on the prostate specific deletion of the *Pten* and/or *Trp53* tumor suppressor genes that are also altered in human prostate cancer [17,18,23]. Tumors that develop in these mice progress in a stage-specific manner, similar to humans, and develop disease-specific features, such CRPC.

We previously compared the antitumor activity of various anti-AR therapies, including apalutamide, in the mouse *Pten*-deficient CNPC model [12]. However, in this study, we have focused on apalutamide and broadened our analysis to include a thorough evaluation of its antitumor activity in the context of *Pten*-deficient CNPC and CRPC in both early-stage and late-stage disease settings, as well as combination therapy with AKT inhibition. We also undertook several experimental approaches to delineate the interactions between apalutamide therapy, AR and the signal pathways essential for prostate cancer cell survival, and have identified the targets as potential causes for resistance to apalutamide that could be further studied and potentially exploited to counter cancer cell survival.

Our findings show that apalutamide alone potently inhibited CNPC in an early-stage model and prolonged survival in late-stage disease. We previously showed that androgen withdrawal promotes phenotypic plasticity by heterogeneously activating multiple compensatory survival pathways that are induced as acute responses to strong AR suppression [17]. In our model, AR responses to apalutamide in CNPC are not as strong as those from surgical castration, and thus may not activate compensatory survival pathways to the same degree as androgen withdrawal. We failed to see any considerable antitumor activity from apalutamide in the early-stage CRPC model. This result was most likely due to a lower dependency of AR in these tumors compared to CNPC. However, in the late-stage *Pten/Trp53*-DKO CRPC model, mice treated with apalutamide were still able to draw a moderate survival benefit. It was also interesting to note that, as in CNPC, CRPC mice treated with apalutamide showed that in tumor regions associated with Ki67-positive cancer cells also expressed higher levels of p-S6 and p-PRAS40. Upregulation of AKT and its targets after AR inhibition has been reported [13]. Our molecular profiling studies in *Pten*-deficient mice have shown that AKT and STAT3 are implicated with castration resistance from androgen deprivation, although upregulation of these survival pathways is heterogenous within the individual tumors [17]. However, in this study, while p-PRAS40 was constitutively expressed regardless of AR status, p-S6 was co-expressed in regions of cells also expressing nuclear AR. Moreover, tumor regions containing KI67-positive cells with no nuclear AR were also positive for p-STAT3pY705. These results suggest that extensive crosstalk networks independent of AR are occurring in *Pten*-deficient CRPC and may be in part contributing to limit the efficacy of apalutamide.

In human prostate cancer, the TP53 gene mutation is a late molecular event in the progression of prostate cancer and is associated with metastasis, loss of differentiation and the transition to CRPC [24]. In our model, inactivation of both PTEN and P53 produces a more aggressive lethal phenotype that is characterized by accelerated growth and decreased survival [18]. Loss of P53 has been shown to decrease the AR levels in human prostate cancer cells in vitro [25,26]. TP53 alterations have been associated with shorter clinical responses to anti-AR with enzalutamide and abiraterone therapy [27]. In our study, we were still able to see some survival benefit when using apalutamide as monotherapy in both the CNPC and CRPC settings. In Phase III clinical trials (TITAN and SPARTAN), treatments with apalutamide have prolonged the survival of patients with metastatic CSPC and nonmetastatic CRPC [28,29].

Various studies have also reported the therapeutic benefits of using combination strategies consisting of AKT/mTOR inhibitors and next-generation anti-androgens in xenograft and transgenic models [30,31]. Here, we examined the combination of apalutamide and GSK690693 on mouse *Pten*-deficient cancer in vitro and in vivo. This treatment combination showed strong potential in vitro, demonstrating strong synergy in both CNPC and CRPC mouse prostate cancer cell lines with loss of PTEN function. Synergy was observed in the *Pten/Trp53*-deficient prostate cancer cell line, although to a lesser degree than the *Pten*-deficient cell with wildtype *Trp53*. In the early-stage intervention models, we did not see appreciable improvement after adding GSK690693 to apalutamide. However, in the CNPC setting, a higher proportion of mice receiving the combination treatment had tumor burden reductions greater than 25% compared apalutamide alone. However, the late-stage model failed to demonstrate any benefit from the combination therapy. Our findings highlight the importance of using autochthonous models to evaluate therapeutic responses. It is possible that this drug combination or formulation could limit the therapeutic efficiency treatment combination, and therefore other therapeutic combinations should be further explored. It is possible that in such an instance, the tumor microenvironment likely played a major role in blunting the combination effect.

Our molecular profiling studies revealed increased PIM-1 expression in cancer cells after treatment with apalutamide or GSK690693 and were further increased in tumors from mice treated with combination therapy. These patterns of increased PIM-1 expression were more pronounced in CRPC. Therefore, we speculate that PIM-1 is implicated in limiting the therapeutic activity of apalutamide and apalutamide/GSK690693 in our models. PIM-1 a is serine threonine kinase having targets that overlap those of AKT and as a result is implicated in mediating resistance to AKT inhibition [32]. PIM-1 is regulated by STAT3 and is overexpressed in high-grade prostatic intraepithelial neoplasia and is overexpressed in 50% of human prostate cancers [33,34]. Transgenic models have shown that co-deletion of *Pten* and *Trp53* in mouse prostate cancer cells enhances IL6 secretion, which then drives STAT3/MYC activation [35]. PIM-1 can phosphorylate AR in CRPC, meaning it can modulate AR function in the absence of androgens [36]. Results from our study further implicate PIM-1 as a key molecule involved in therapeutic resistance to AR and AKT inhibition and provide additional evidence to warrant further investigation of PIM-1 in prostate cancer.

## 4. Materials and Methods

### 4.1. Treatment Compounds

Apalutamide (catalog no. HY-16060) and GSK690693 (catalog no. HY-10249) were purchased from MedChemExpress (Monmouth Junction, NJ, USA). For in vivo oral (p.o.) administration of apalutamide, a 10 mg/mL emulsion was prepared in 1% carboxymethylcellulose (CMC), 0.1% Tween 80 and 5% DMSO in dH2O. GSK690693 was prepared as a stock solution (3 mg/mL) in 4% DMSO, 40% 2-hydroxypropyl-β-cyclodextrin (2-HPBC) pH 6.0 in dH2O for intraperitoneal (i.p.) administration. Compounds were administered orally using a solution prepared freshly each week. The base volume was 100 µL per dose for every 30-g mouse and the dosing volumes were adjusted as necessary.

### 4.2. Animals

Conditional prostate-specific *PSA^Cre^: Pten^flox/flox^ Pten* knockout (*Pten*-KO) and *PSA^Cre^: Pten^flox/flox^:Trp53^flox/flox^ Pten/P53* double knockout (*Pten/Trp53*-DKO) mice were used in this study, and have been described previously [17,18]. Animals were housed at the Kindai University Faculty of Medicine Animal Facility in accordance with institutional guidelines and procedures were carried out in compliance with the standards for use of laboratory animals. This study was approved by the Institutional Review Committee at Kindai University Faculty of Medicine.

### 4.3. Efficacy Studies

Aged-matched mice were randomized at weaning using the stratified random sampling method and housed in cages containing 2–4 mice. When mice reached the appropriate age, cages of the age-matched mice were randomly assigned to treatment cohorts. For early-stage CNPC intervention studies, *Pten*-KO mice aged between 18 and 32 weeks were treated for four weeks with the vehicle (1% CMC/0.1% Tween 80/5% DMSO), apalutamide (30 mg/kg, p.o. 5 times/week), GSK690693 (10 mg/kg, i.p. 5 times/week) or were surgically castrated, as described previously [12]. For the early-stage CRPC intervention studies, 12-week-old mice were surgically castrated and pharmacological treatments were started at 18 weeks of age. Mice were euthanized four hours after the last treatment. Genitourinary tracts (GUTs) were removed en bloc, weighed and imaged. The dorsolateral and ventral prostate lobes were then dissected as a unit bilaterally, weighed, portioned and flash frozen in liquid nitrogen, preserved in RNAlater solution (Invitrogen, Camarillo, CA, USA), or fixed overnight in 10% neutral buffered formalin. Tumor burden was defined as the percent prostate weight relative to bodyweight.

Survival studies were conducted on *Pten/Trp53*-DKO mice with palpable tumors as a model of advanced prostate cancer, as previously described [18]. In this model, *Pten/Trp53-*DKO mice were palpated and were surgically castrated when the prostate tumor reached 0.5 cm in diameter. Mice were palpated weekly and randomized to treatment cohorts using permuted block randomization when tumors doubled in size or 2 weeks after castration, whichever occurred first. Mice were monitored daily, and tumor growth was monitored weekly. Mice were sacrificed when the tumor reached 3 cm in maximum diameter, experienced >20% bodyweight loss from baseline or exhibited abnormal behavior/demeanor, distended abdomen or hematuria. Experimental endpoints were overall survival, cumulative survival, time to progression and post progression survival and relative tumor growth rates. Genitourinary tract weight was used to measure tumor burden. Relative tumor growth rates were inferred using final tumor volume and survival times using exponential regression analysis, *y = ae^ax^* (assuming *a* > 0).

### 4.4. Histology and Immunohistochemistry (IHC)

Formalin-fixed, paraffin-embedded tissues were sectioned and stained with hematoxylin and eosin (H&E) using standard methods. The primary antibodies, concentration and specific pretreatments are summarized in Supplementary Table S1. Briefly, serial sections were incubated with the primary antibody overnight at 4 °C and then rinsed and stained using the ABC kit (Vector Laboratories, Burlingame, CA, USA) following the manufacturer’s instructions. Slides were developed in DAB developed in DAB (Invitrogen, Camarillo, CA, USA) and counterstained with hematoxylin. Quantitative IHC was performed on slide captures representing 4–10 representative regions per sample and percent positivity or H scores were calculated with the QuPath v0.2.0-m4 image analysis software [37].

### 4.5. Western Blot Analysis

Protein extracts were prepared from the snap-frozen prostate tumors using RIPA buffer with HALT protease and phosphatase inhibitors (Thermo Scientific, Rockford, IL, USA). SDS-gel electrophoresis was performed on Novex 4–20% Tris-Glycine Gels (Invitrogen, Camarillo, CA, USA) and the proteins were transferred onto Immobilon-P PVDF membranes (MilliporeSigma, Burlington, MA, USA). Membranes were blocked with 3% BSA-TBST (50 mM Tris-HCl, 150 mM NaCl, Tween-20) and probed with the primary antibodies listed in Supplementary Table S1. Blots were then developed in ECL Prime solution (GE Healthcare, Piscataway, NJ, USA) and images were captured using the LAS-4010 ImageQuant imaging system (GE Healthcare, Piscataway, NJ, USA). Membrane stripping was achieved by subjecting them to SDS/β-mercaptoethanol solution (62.5 mM tris (pH 6.8), 2% SDS, and 100 mM β-mercaptoethanol) at 50 °C for 30 min. Stripped membranes were washed in TBST buffer, blocked and re-probed with additional primary antibodies as needed. Semi-quantitative densitometric analyses were made with ImageJ analysis software (https://imagej.nih.gov/ij/, accessed on 17 May 2020) using GAPDH as a loading control [38].

### 4.6. RNA Extraction and qRT-PCR Analysis

The prostate samples used in the qRT-PCR analyses were preserved in RNAlater storage solution (Thermo Scientific, Rockford, IL, USA). Total RNA was extracted from the prostate tissues and purified using the RNeasy Mini Kit (Qiagen, Germantown, MD, USA) following the manufacturer’s instructions. cDNA was synthesized using the PrimeScript First Strand cDNA Synthesis Kit (Takara, Shiga, Japan) following the instructions provided. Primers for the genes used in the analyses are shown in Supplementary Table S2 and have been previously reported [12]. cDNA concentration was measured by qRT–PCR using the SYBR Premix Ex Taq II Kit (Takara, Shiga, Japan) and relative changes in gene expression levels were determined using the 2^−ΔΔCt^ method using GAPDH as an internal control.

### 4.7. In Vitro Studies

Mouse prostate cancer cell lines established from *Pten*-deficient CNPC 7109-G4 and CRPC, 2945-F12 and *Pten/Trp53*-deficient CNPC, 3902-A1 and CRPC, 4522-B8 were used and have been previously described [12,39]. Cells were maintained at 37 °C under 5% CO2 using RPMI media supplemented with 10% FBS and 1% penicillin/streptomycin. Apalutamide and GSK690693 were prepared as stock dilutions in DMSO. For determination of the effective doses resulting in 50% growth inhibition (ED_50_), cells were incubated in media treated with serially diluted concentrations of apalutamide or GSK690693 for 96 h. Cell viability was determined using the crystal violet cell viability assay reading absorbance at 595 nm. Synergy was determined by exposing cells for 96 h to media treated with constant ratio combinations ED_50_ of apalutamide and GSK690693. The synergistic effect was calculated according to the Chou–Talalay method with CompuSyn v1.0 software.

### 4.8. Plotting and Statistical Analysis

Unless otherwise specified, statistical analyses were performed using SigmaPlot v13.0. Hierarchical clustering and heatmaps were generated with Morpheus, https://software.broadinstitute.org/morpheus, accessed on 12 January 2021 [40]. Clustered correlation plots were generated in R (https://www.r-project.org/, accessed on 1 October 2020) using the R packages “corrplot” and “hclust” and partitioning around medoids (PAM) K-medoids clustering plots were generated with the R packages ‘factoextra’ and “cluster” [41]. FreeViz multivariate plots were generated with Orange [42]. *p* values less than 0.05 were considered statistically significant.

## 5. Conclusions

In summary, we have used faithful preclinical mouse models of the prostate to evaluate and profile the activity of apalutamide in the context of *Pten*-deficient prostate cancer during different stages of the disease process. This study also provides data that can be utilized to further study the utility of additional combinations with apalutamide. In conclusion, our findings show that apalutamide can attenuate *Pten*-deficient prostate cancer in a context-specific manner.

## Figures and Tables

**Table 1:** Summary of the mean effective dose for 50% growth inhibition (ED_50_) of apalutamide and GSK-690693 and the combination index (CI) at ED_50_ for mouse prostate cancer cell lines.

Cell Line	Description	Apalutamide		GSK690693		Combination Index	
		Mean ED_50_	SE	Mean ED_50_	SE	Mean CI	SE
		(µM)		(µM)			
7109-G4	CNPC *Pten*-deficient tumor	28.70	8.16	9.18	2.57	0.585	0.053
2945-F12	CRPC *Pten-*deficient tumor	56.17	5.16	1.28	0.33	0.628	0.046
3902-A1	CNPC *Pten/Trp53*-deficient tumor	41.77	5.59	1.87	0.69	0.73	0.063
4522-B8	CRPC *Pten/Trp53*-deficient tumor	36.37	4.1	7.79	1.33	0.791	0.053

Drug–drug interactions were considered synergistic (CI < 0.9), additive (CI = 0.9–1.0) and antagonistic (CI > 1).

## Data Availability

The data presented in this study are available on request from the corresponding author.

## Supplementary Materials

The following are available online at https://www.mdpi.com/article/10.3390/cancers13163975/s1. Figure S1. Influence of apalutamide on mice with advanced *Pten*-deficient castration-naïve prostate (CNPC). Figure S2. Molecular correlates of apalutamide in *Pten*-deficient castration-naïve prostate cancer (CNPC). Figure S3. Effects of apalutamide on mice with *Pten*-deficient castration-resistant prostate (CRPC). Figure S4. Molecular correlates of apalutamide in *Pten*-deficient castration-resistant prostate cancer (CRPC). Figure S5. Visualization of the Western blot correlates of apalutamide in *Pten*-deficient castra-tion-resistant prostate cancer (CRPC). Table S1. Antibody list: antibodies for immunohistochemistry. Table S2. List of PCR primers used for qRT-PCR.

## References

[1] Joseph J.D., Lu N., Qian J., Sensintaffar J., Shao G., Brigham D., Moon M., Maneval E.C., Chen I., Darimont B. (2013). A clinically relevant androgen receptor mutation confers resistance to second-generation antiandrogens enzalutamide and ARN-509. Cancer Discov..

[2] Antonarakis E.S., Lu C., Wang H., Luber B., Nakazawa M., Roeser J.C., Chen Y., Fedor H.L. (2014). AR-V7 and resistance to enzalutamide and abiraterone in prostate cancer. N. Eng. J. Med..

[3] Ferraldeschi R., Welti J., Luo J., Attard G., De Bono J.S. (2015). Targeting the androgen receptor pathway in castration-resistant prostate cancer: Progresses and prospects. Oncogene.

[4] Hanahan D., Weinberg R.A. (2011). Hallmarks of cancer: The next generation. Cell.

[5] Taylor B.S., Schultz N., Hieronymus H., Gopalan A., Xiao Y., Carver B.S., Arora V.K., Kaushik P., Cerami E., Reva B. (2010). Integrative Genomic Profiling of Human Prostate Cancer. Cancer Cell.

[6] Sircar K., Yoshimoto M., Monzon F.A., Koumakpayi I.H., Katz R.L., Khanna A., Alvarez K., Chen G., Darnel A.D., Aprikian A.G. (2009). PTEN genomic deletion is associated with p-Akt and AR signalling in poorer outcome, hormone refractory prostate cancer. J. Pathol..

[7] Ferraldeschi R., Rodrigues D.N., Riisnaes R., Miranda S., Figueiredo I., Rescigno P., Ravi P., Pezaro C., Omlin A., Lorente D. (2015). PTEN protein loss and clinical outcome from castration-resistant prostate cancer treated with abiraterone acetate. Eur. Urol..

[8] Krohn A., Diedler T., Burkhardt L., Mayer P.-S., De Silva C., Meyer-Kornblum M., Kötschau D., Tennstedt P., Huang J., Gerhauser C. (2012). Genomic deletion of PTEN is associated with tumor progression and early PSA recurrence in ERG fusion-positive and fusion-negative prostate cancer. Am. J. Pathol..

[9] Nan B., Snabboon T., Unni E., Yuan X.J., Whang Y., Marcelli M. (2003). The PTEN tumor suppressor is a negative modulator of AR transcriptional activity. J. Mol. Endocrinol..

[10] Watson P.A., Chen Y.F., Balbas M.D., Wongvipat J., Socci N.D., Viale A., Kim K., Sawyers C.L. (2010). Constitutively active androgen receptor splice variants expressed in castration-resistant prostate cancer require full-length androgen receptor. Proc. Natl. Acad. Sci. USA.

[11] Liang M., Adisetiyo H., Liu X., Liu R., Gill P., Roy-Burman P., Jones J.O., Mulholland D.J. (2015). Identification of androgen receptor splice variants in the pten deficient murine prostate cancer model. PLoS ONE.

[12] De Velasco M.A., Kura Y., Sakai K., Hatanaka Y., Davies B.R., Campbell H., Klein S., Kim Y., MacLeod A.R., Sugimoto K. (2019). Targeting castration-resistant prostate cancer with androgen receptor antisense oligonucleotide therapy. JCI Insight.

[13] Carver B.S., Chapinski C., Wongvipat J., Hieronymus H., Chen Y., Chandarlapaty S., Arora V.K., Le C., Koutcher J., Scher H. (2011). Reciprocal Feedback Regulation of PI3K and Androgen Receptor Signaling in PTEN-Deficient Prostate Cancer. Cancer Cell.

[14] Mulholland D.J., Tran L.M., Li Y., Cai H., Morim A., Wang S., Plaisier S., Garraway I.P., Huang J., Graeber T.G. (2011). Cell autonomous role of PTEN in regulating castration-resistant prostate cancer growth. Cancer Cell.

[15] Choucair K., Ejdelman J., Brimo F., Aprikian A., Chevalier S., Lapointe J. (2012). PTEN genomic deletion predicts prostate cancer recurrence and is associated with low AR expression and transcriptional activity. BMC Cancer.

[16] Clegg N.J., Wongvipat J., Joseph J.D., Tran C., Ouk S., Dilhas A., Chen Y., Grillot K., Bischoff E.D., Cai L. (2012). ARN-509: A novel antiandrogen for prostate cancer treatment. Cancer Res..

[17] De Velasco M.A., Tanaka M., Yamamoto Y., Hatanaka Y., Koike H., Nishio K., Yoshikawa K., Uemura H. (2014). Androgen deprivation induces phenotypic plasticity and promotes resistance to molecular targeted therapy in a PTEN-deficient mouse model of prostate cancer. Carcinogenesis.

[18] De Velasco M.A., Kura Y., Yoshikawa K., Nishio K., Davies B.R., Uemura H. (2016). Efficacy of targeted AKT inhibition in genetically engineered mouse models of PTEN-deficient prostate cancer. Oncotarget.

[19] Rybak A.P., Bristow R.G., Kapoor A. (2015). Prostate cancer stem cells: Deciphering the origins and pathways involved in prostate tumorigenesis and aggression. Oncotarget.

[20] Shorning B.Y., Dass M.S., Smalley M.J., Pearson H.B. (2020). The PI3K-AKT-mTOR pathway and prostate cancer: At the crossroads of AR, MAPK, and WNT signaling. Int. J. Mol. Sci..

[21] Liu C., Zhu Y., Lou W., Cui Y., Evans C.P., Gao A.C. (2014). Inhibition of constitutively active Stat3 reverses enzalutamide resistance in LNCaP derivative prostate cancer cells. Prostate.

[22] Cen B., Mahajan S., Wang W., Kraft A.S. (2013). Elevation of receptor tyrosine kinases by small molecule AKT inhibitors in prostate cancer is mediated by Pim-1. Cancer Res..

[23] Quigley D.A., Dang H.X., Zhao S.G., Lloyd P., Aggarwal R., Alumkal J.J., Foye A., Kothari V., Perry M., Bailey A.M. (2018). Genomic Hallmarks and Structural Variation in Metastatic Prostate Cancer. Cell.

[24] Navone N.M., Troncoso P., Pisters L.L., Goodrow T.L., Palmer J.L., Nichols W.W., Von Eschenbach A.C., Conti C.J. (1993). P53 protein accumulation and gene mutation in the progression of human prostate carcinoma. JNCI.

[25] Cronauer M.V., Schulz W.A., Burchardt T., Ackermann R., Burchardt M. (2004). Inhibition of p53 function diminishes androgen receptor-mediated signaling in prostate cancer cell lines. Oncogene.

[26] Alimirah F., Panchanathan R., Chen J., Zhang X., Ho S.-M., Choubey D. (2007). Expression of androgen receptor is negatively regulated by p53. Neoplasia.

[27] Abida W., Cyrta J., Heller G., Prandi D., Armenia J., Coleman I., Cieslik M., Benelli M., Robinson D., Van Allen E.M. (2019). Genomic correlates of clinical outcome in advanced prostate cancer. Proc. Natl. Acad. Sci. USA.

[28] Small E.J., Saad F., Chowdhury S., Oudard S., Hadaschik B.A., Graff J.N., Olmos D., Mainwaring P.N., Lee J.Y., Uemura H. (2020). Final survival results from SPARTAN, a phase III study of apalutamide (APA) versus placebo (PBO) in patients (pts) with nonmetastatic castration-resistant prostate cancer (nmCRPC). J. Clin. Oncol..

[29] Chi K.N., Agarwal N., Bjartell A., Chung B.H., Gomes A.J.P.D.S., Given R., Given R., Soto Á.J., Merseburger A.S., Özgüroglu M. (2019). Apalutamide for Metastatic, Castration-Sensitive Prostate Cancer. N. Eng. J. Med..

[30] Marques R.B., Aghai A., de Ridder C.M., Stuurman D., Hoeben S., Boer A., Ellston R., Barry S.T., Davies B.R., Trapman J. (2015). High efficacy of combination therapy using PI3K/AKT inhibitors with androgen deprivation in prostate cancer preclinical models. Eur. Urol..

[31] Toren P., Kim S., Cordonnier T., Crafter C., Davies B.R., Fazli L., Gleave M.E., Zoubeidi A. (2015). Combination AZD5363 with enzalutamide significantly delays enzalutamide-resistant prostate cancer in preclinical models. Eur. Urol..

[32] Warfel N.A., Kraft A.S. (2015). PIM kinase (and Akt) biology and signaling in tumors. Pharm. Ther..

[33] Valdman A., Fang X., Pang S.-T., Ekman P., Egevad L. (2004). Pim-1 expression in prostatic intraepithelial neoplasia and human prostate cancer. Prostate.

[34] Dhanasekaran S.M., Barrette T.R., Ghosh D., Shah R., Varambally S., Kurachi K., Pienta K., Rubin M., Chinnaiyan A.M. (2001). Delineation of prognostic biomarkers in prostate cancer. Nature.

[35] Nowak D.G., Cho H., Herzka T., Watrud K., Demarco D.V., Wang V., Senturk S., Fellmann C., Ding D., Beinortas T. (2015). MYC drives Pten/Trp53-deficient proliferation and metastasis due to IL6 secretion and AKT suppression via PHLPP2. Cancer Discov..

[36] Ha S., Iqbal N., Mita P., Ruoff R., Gerald W.L., Lepor H., Taneja S.S., Lee P., Melamed J., Garabedian M.J. (2013). Phosphorylation of the androgen receptor by PIM1 in hormone refractory prostate cancer. Oncogene.

[37] Bankhead P., Loughrey M.B., Fernández J.A., Dombrowski Y., McArt D., Dunne P.D., McQuaid S., Gray R.T., Murray L.J., Coleman H.G. (2017). QuPath: Open-source software for digital pathology image analysis. Sci. Rep..

[38] ImageJ Image Processing and Analysis in Java. https://imagej.nih.gov/ij/.

[39] Takao A., Yoshikawa K., Karnan S., Ota A., Uemura H., De Velasco M.A., Kura Y., Suzuki S., Ueda R., Nishino T. (2018). Generation of PTEN-knockout (−/−) murine prostate cancer cells using the CRISPR/Cas9 system and comprehensive gene expression profiling. Oncol. Rep..

[40] Morpheus. https://software.broadinstitute.org/morpheus.

[41] R: The R Project for Statistical Computing. https://www.r-project.org/.

[42] Demšar J., Curk T., Erjavec A., Gorup Č., Hočevar T., Milutinovič M., Možina M., Polajnar M., Toplak M., Starič A. (2013). Orange: Data Mining Toolbox in Python. J. Mach. Learn. Res..

