# Supplementary material for: Context-Specific Efficacy of Apalutamide Therapy in Preclinical Models of Pten-Deficient Prostate Cancer
Source: Cancers (Basel). 2021 Aug 6;13(16):3975. doi: 10.3390/cancers13163975 (PMC8391912; doi:10.3390/cancers13163975)
Supplement: Supplementary file 1 [file cancers-13-03975-s001.zip › cancers-1293015_supplementary revised.pdf]

cancers

MDPI

Article

Context-Specific Efficacy of Apalutamide Therapy in Preclinical Models of *Pten*-Deficient Prostate Cancer

Marco A. De Velasco, Yurie Kura, Naomi Ando, Noriko Sako, Eri Banno, Kazutoshi Fujita, Masahiro Nozawa, Kazuhiro Yoshimura, Kazuko Sakai, Kazuhiro Yoshikawa, Kazuto Nishio and Hirotsugu Uemura

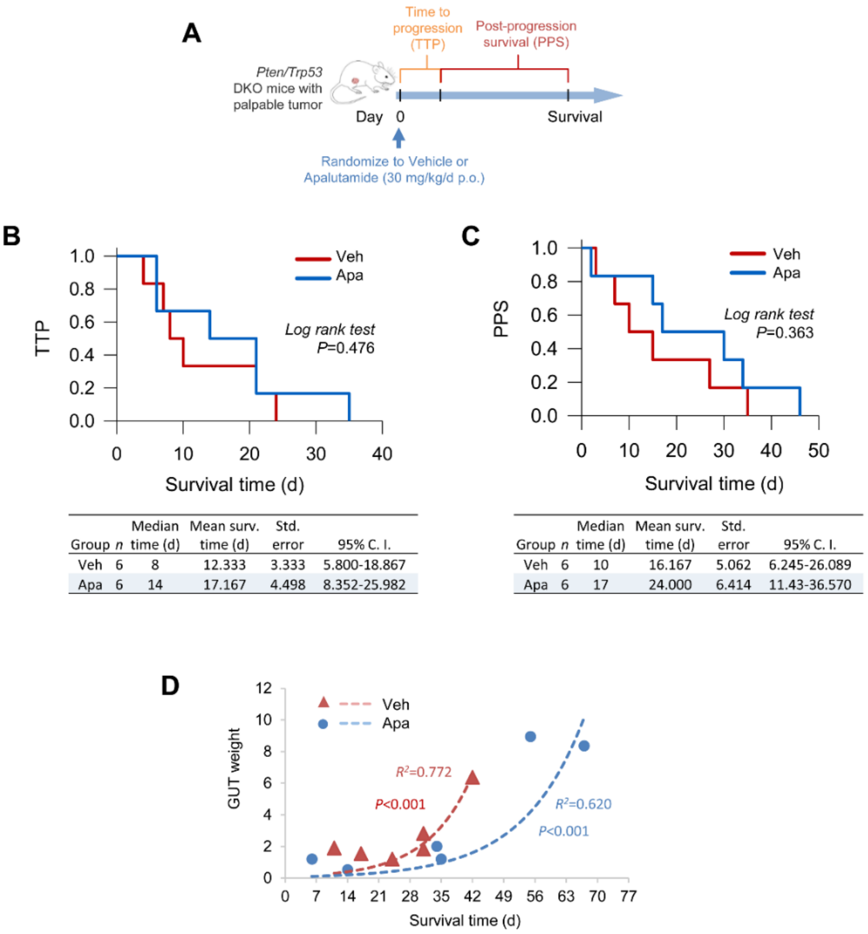

Figure S1. Influence of apalutamide on mice with advanced *Pten*-deficient castration-naïve prostate (CNPC). PSA^{Cre};Pten$^{loxP/loxP}$/Trp53$^{loxP/loxP}$ (DKO) mice were randomized to the vehicle (Veh) or apalutamide (Apa) when palpable tumors reached 5 mm. (**A**) Schema illustrating the experimental design and treatment endpoints. Kaplan–Meier plots showing the survival curves for time to progression (TTP) and (**B**) post-progression survival (PPS) (**C**) of DKO mice treated with apalutamide (30 mg/kg, p.o. 5times/week) or the vehicle. (**D**) Comparison of the growth curves of tumors inferred from the final genitourinary tract (GUT) and survival time using the exponential growth formula. *p* values were calculated for the t statistic, t = 39.728 and 26.74 for Veh and Apa, respectively.

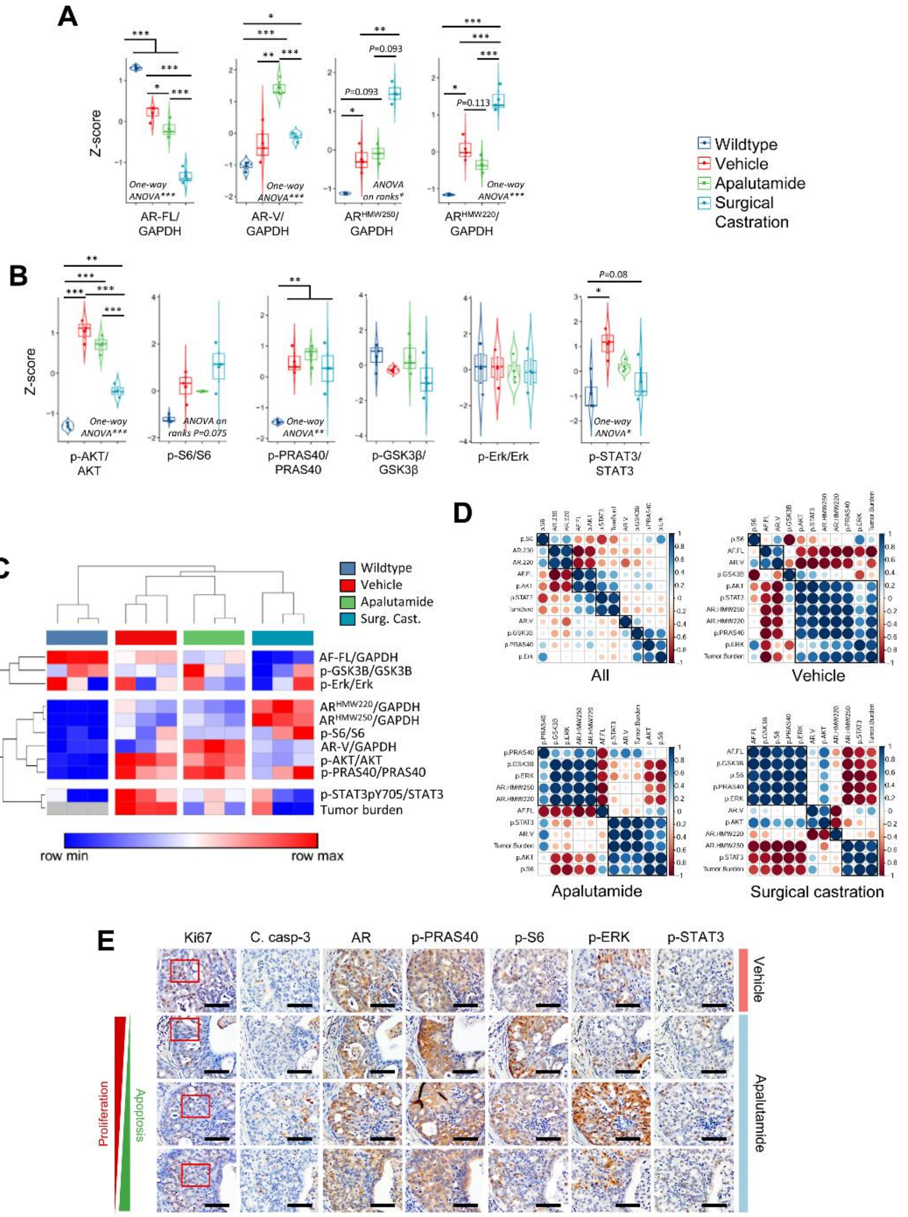

Figure S2. Molecular correlates of apalutamide in *Pten*-deficient castration-naïve prostate cancer (CNPC). (**A**,**B**) Plots of normalized protein expression data from semi-quantitative densitometry analysis of the Western blots in Figure 2B. Horizontal bars represent the mean ± SE, center lines show the medians and box limits indicate the 25th and 75th percentiles, and the violin plots show the distribution with circles representing individual samples; $n = 3$ mice/group. Significance was calculated using the Student–Newman–Keuls post hoc test for individual comparisons, upon significant one-way ANOVA or ANOVA on ranks as indicated (* $p < 0.05$, ** $p < 0.01$, and *** $p < 0.001$). (**C**) Heatmap and unsupervised hierarchical clustering of the z-score-transformed data of the semiquantitative Western blot data and tumor burden using average linkage and Euclidean distance. (**D**) Correlation plots of the densitometric data clustered using the median distance. Colored scales represent the Pearson's correlation coefficient and circle size corresponds to correlation strength. (**E**) Low magnification image of Figure 2E. Corresponding regions are marked with a red bounding square; scale bars = 100 μm.

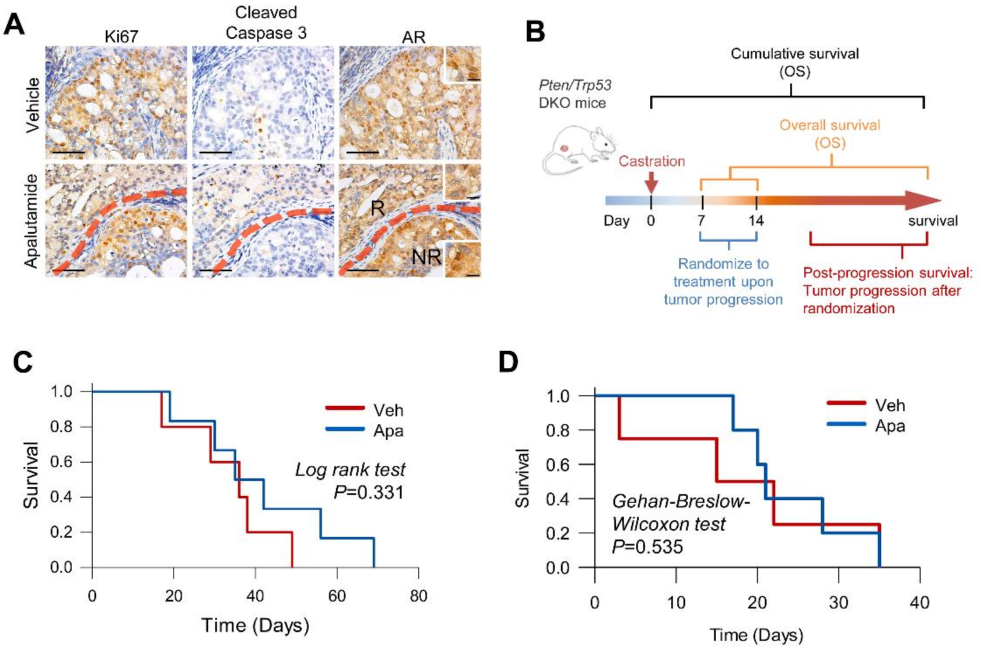

Group	*n*	Median time (d)	Mean surv. time (d)	Std. error	95% C. I.
Veh	5	36	33.800	5.286	23.440-44.160
Apa	6	35	41.833	7.409	27.312-56.355

Group	*n*	Median time (d)	Mean surv. time (d)	Std. error	95% C. I.
Veh	4	15	18.750	6.688	5.642-31.858
Apa	5	21	24.200	3.247	17.837-30.563

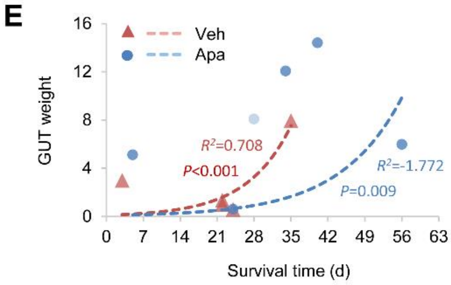

Figure S3. Effects of apalutamide on mice with *Pten*-deficient castration-resistant prostate (CRPC). (**A**) Representative photomicrographs of immunostained mouse CRPC treated with the vehicle (Veh) or apalutamide (Apa, 30 mg/kg, p.o. 5 times/week) for four weeks. Serial sections were stained with antibodies against Ki67, cleaved caspase-3 and androgen receptor (AR). Treatment responsive (R) and non-responsive (NR) regions in the apalutamide-treated tumor are demarcated with a dashed line. Scale bars = 100 μm and the insert scale bar = 10 μm. (**B**) Schema illustrating the experimental design of advanced prostate cancer in PSA^{Cre};$Pten^{loxP/loxP}$/$Trp53^{loxP/loxP}$ (DKO) mice and the endpoints used to assess the therapeutic benefit of apalutamide. Kaplan–Meier plots showing the survival curves for cumulative survival and (**C**) post-progression survival (PPS) (**D**) of DKO mice treated with apalutamide (30 mg/kg, p.o. 5times/week) or the vehicle. (**E**) Comparison of the growth curves of tumors inferred from the final genitourinary tract (GUT) and the survival time using the exponential growth formula. *p* values were calculated for the t statistic, t = 24.721 and 6.998 for Veh and Apa, respectively.

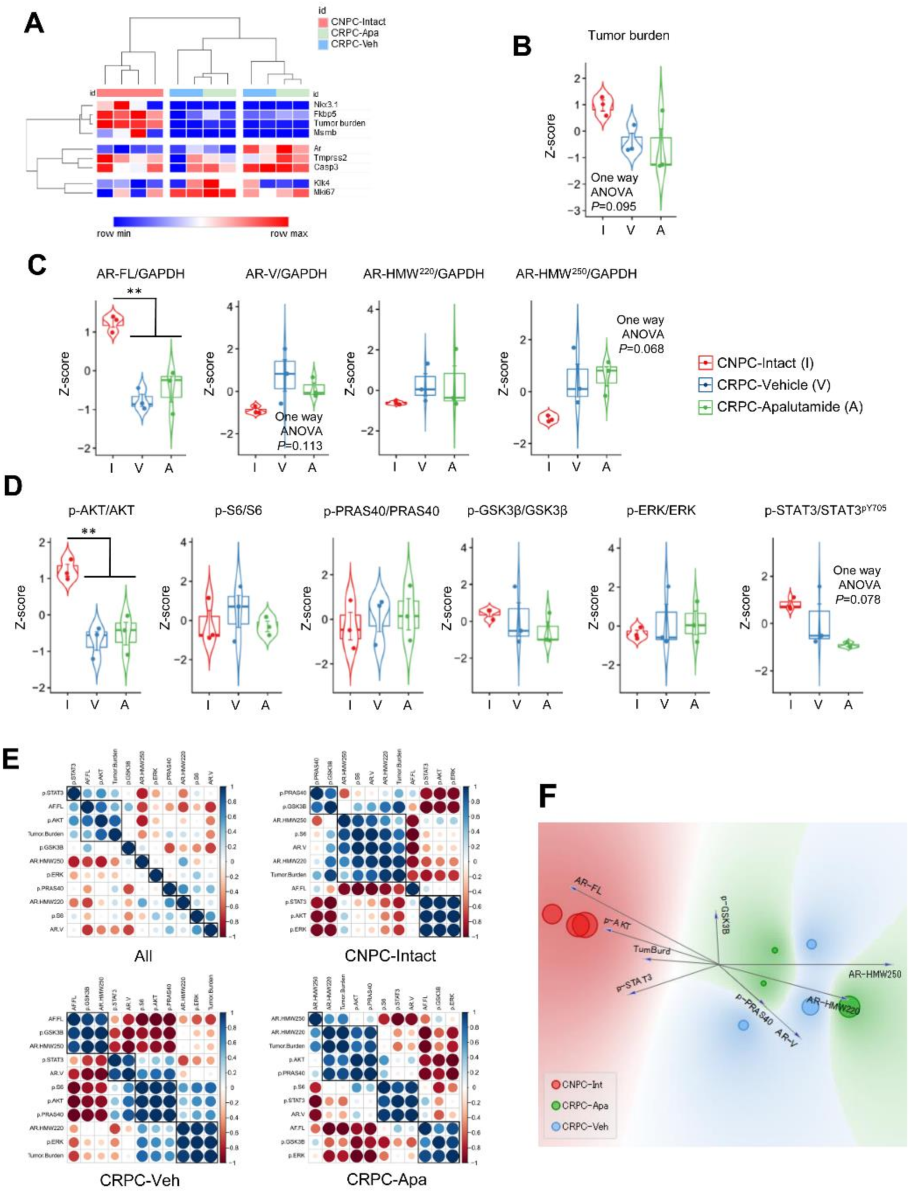

Figure S4. Molecular correlates of apalutamide in *Pten*-deficient castration-resistant prostate cancer (CRPC). (**A**) Heatmap of the z-score transformed data of the tumor burden and qRT-PCR mRNA expression data of Ar and AR target genes in intact CNPC (I) and CRPC treated with the vehicle (V) or apalutamide (**A**); *n* = 4 mice/group. Dendrograms show unsupervised hierarchical clustering using the average linkage and Euclidean distance. (**B**) Comparison of tumor burden in tumors Figure 4. B (intact CNPC (I), CRPC treated with vehicle (V) or apalutamide (**A**); *n* = 3 mice/group). (**C**,**D**) Plots of the normalized protein expression data from the semi-quantitative densitometry analysis of the Western blots in Figure 4B. Horizontal bars represent the mean ± SE, center lines show the medians and box limits indicate the 25th and 75th percentiles, and the violin plots show the distribution with circles representing individual samples; *n* = 3 mice/group. Significance was calculated with the Student–Newman–Keuls post hoc test for individual comparisons, upon a significant *one-way ANOVA* or *ANOVA on ranks* as indicated (** $p < 0.01$). (**E**) Correlation plots of the densitometric data clustered using the median distance. Colored scales represent the Pearson's correlation coefficient and the circle size corresponds to the correlation strength. (**F**) Freeviz plot showing the visual multivariate correlates to treatment. Length of the vectors indicate the strength of association and the marker size corresponds to tumor burden.

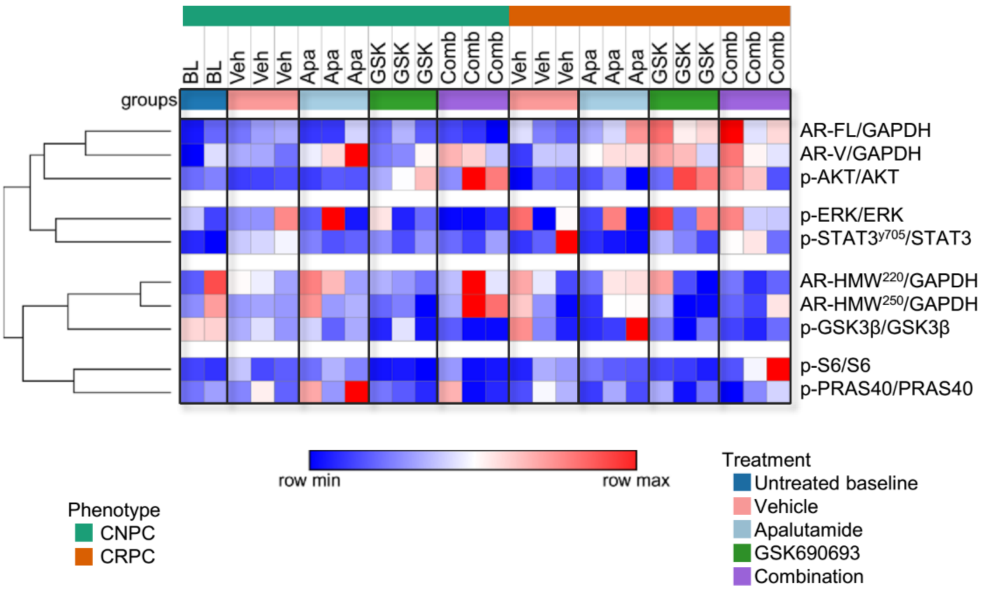

Figure S5. Visualization of the Western blot correlates of apalutamide in *Pten*-deficient castration-resistant prostate cancer (CRPC). Heatmap from normalized protein expression data from semi-quantitative densitometry analysis of the Western blots in Figure 7A. Protein expression levels of full length AR (AR-FL), AR splicing variant (AR-V) and high-molecular weight AR (AR-HMW220 and AR-HMW250) were normalized to GAPDH. Phosphorylated signal molecules were normalized to total protein levels. Relative values were normalized to the corresponding vehicle-treated group in each castration-naïve (CNPC) or castration-resistant prostate cancer (CRPC); n = 2–3 mice/group. Dendrograms show unsupervised hierarchical clustering using average linkage and Euclidean distance.

Table S1. Antibody list: antibodies for immunohistochemistry

Marker	Source	Catalogue Number	Antigen Retrieval	Dilution	Secondary Antibody	Reference
Ki67	Thermo Fisher Scientific	RB-9043	Dako ARS	1:250	Rabbit	https://www.antibodypedia.com/gene/741/MKI67/antibody/630324/PA5-16446 (accessed on: 10 May 2019)
Cleaved Caspase-3	Cell Signaling	9661	Dako ARS	1:400	Rabbit	https://www.antibodypedia.com/gene/1222/CASP3/antibody/108063/9661 (accessed on: 10 May 2019)
Androgen receptor	Thermo Fisher Scientific	RB-9030	Dako ARS	1:100	Rabbit	[12]
P-S6	Cell Signaling	2211	Dako ARS	1:100	Rabbit	https://www.antibodypedia.com/gene/3412/RPS6/antibody/105615/2211 (accessed on: 10 May 2019)
P-PRAS40	Cell Signaling	13175	Dako ARS	1:100	Rabbit	https://www.antibodypedia.com/gene/32195/AKT1S1/antibody/1286783/13175 (accessed on: 10 May 2019)
p-ERK	Cell Signaling	4370	Dako ARS	1:100	Rabbit	[12]
p-STAT3 pY705	Cell Signaling	9145	Dako ARS	1:100	Rabbit	[12]
PIM-1	Cell Signaling	3247	Dako ARS	1:100	Rabbit	https://www.antibodypedia.com/gene/1207/PIM1/antibody/106391/3247 (accessed on: 10 May 2019)
Antibodies for Western Blot						
Androgen receptor	Thermo Fisher Scientific	RB-9030	110	1:1000	Rabbit	[12]
Akt	Cell Signaling	9272	60	1:1000	Rabbit	https://www.antibodypedia.com/gene/135/AKT1/antibody/166716/9272 (accessed on: 10 May 2019)
P-Akt pSer473	Cell Signaling	4060	60	1:1000	Rabbit	https://www.antibodypedia.com/gene/135/AKT1/antibody/106995/4060 (accessed on: 10 May 2019)
S6	Cell Signaling	2217	32	1:1000	Rabbit	https://www.antibodypedia.com/gene/3412/RPS6/antibody/105619/2217 (accessed on: 10 May 2019)
P-S6	Cell Signaling	2211	32	1:1000	Rabbit	https://www.antibodypedia.com/gene/3412/RPS6/antibody/105615/2211 (accessed on: 10 May 2019)
PRAS40	Cell Signaling	2610	40	1:1000	Rabbit	https://www.antibodypedia.com/gene/32195/AKT1S1/antibody/105922/2610 (accessed on: 10 May 2019)
P-PRAS40	Cell Signaling	13175	40	1:1000	Rabbit	https://www.antibodypedia.com/gene/32195/AKT1S1/antibody/1286783/13175 (accessed on: 10 May 2019)

GSK3β	Cell Signaling	9315	46	1:1000	Rabbit	https://www.antibodypedia.com/gene/4266/GSK3B/antibody/107924/9315 (accessed on: 10 May 2019)
p-GSK3β	Cell Signaling	9336	46	1:1000	Rabbit	https://www.antibodypedia.com/gene/4266/GSK3B/antibody/107934/9336 (accessed on: 10 May 2019)
ERK	Cell Signaling	9102	42, 44	1:1000	Rabbit	https://www.antibodypedia.com/gene/1203/MAPK3/antibody/166714/9102 (accessed on: 10 May 2019)
p-ERK	Cell Signaling	4370	42, 44	1:1000	Rabbit	https://www.antibodypedia.com/gene/1203/MAPK3/antibody/167085/4370 (accessed on: 10 May 2019)
STAT3	Cell Signaling	4904	79, 86	1:1000	Rabbit	https://www.antibodypedia.com/gene/660/STAT3/antibody/107536/4904 (accessed on: 10 May 2019)
p-STAT3 pY705	Cell Signaling	9145	79, 86	1:1000	Rabbit	https://www.antibodypedia.com/gene/660/STAT3/antibody/107831/9145 (accessed on: 10 May 2019)
GAPDH	Cell Signaling	2118	37	1:10000	Rabbit	https://www.antibodypedia.com/gene/3923/GAPDH/antibody/105543/2118 (accessed on: 10 May 2019)

DAKO ARS: 20 min steam heating in DAKO Target retrieval solution (S1699, Agilent, Santa Clara, CA, USA).

Table S2. List of PCR primers used for qRT-PCR.

Target	Strand	Sequence	Source
Ar	Forward	CCAGTGGATGGGCTGAAAAAT	PrimerBlast
	Reverse	CCTTGAGCAGGATGTGGGATT	
Casp3	Forward	GACTTGCTCCCATGTATGGTC	qPrimerDepot
	Reverse	ATCAAAGCGCAGTGTCCTG	
Fkbp5	Forward	CCAACAACGAACACCACATC	qPrimerDepot
	Reverse	CGAGGGATACTCAAACCCAA	
Gapdh	Forward	CGAACGGGAAGCTCACTGGCAT	qPrimerDepot
	Reverse	TCAGATGCCTGCTTCACCACCT	
Klk4	Forward	CTTGTATGATCCGGCTGCTG	qPrimerDepot
	Reverse	AGCCAACATGATGGTCACTG	
Mki67	Forward	CCATCTGAGGCAGGGCTATC	PrimerBlast
	Reverse	TTTCATATTTATTGCCAAGATGGGG	
Msmb	Forward	ACAGGTCTTCCCTGGGTTCT	qPrimerDepot
	Reverse	CGTCAATCACCTGCTGTACC	
Nkx3.1	Forward	CACTTGCTAAGTCCCCTGGA	qPrimerDepot
	Reverse	AGTATCCGGCATAGCCCC	
Tmprss2	Forward	CTCCCGTAGCTCTCACTCCA	qPrimerDepot
	Reverse	CCCAACGGAGAAGATGAGAA	
