# Supplementary material for: Context-Specific Efficacy of Apalutamide Therapy in Preclinical Models of Pten-Deficient Prostate Cancer
Source: Cancers (Basel). 2021 Aug 6;13(16):3975. doi: 10.3390/cancers13163975 (PMC8391912; doi:10.3390/cancers13163975)

De Velasco et al.

Full unedited membranes for Figure 2B.

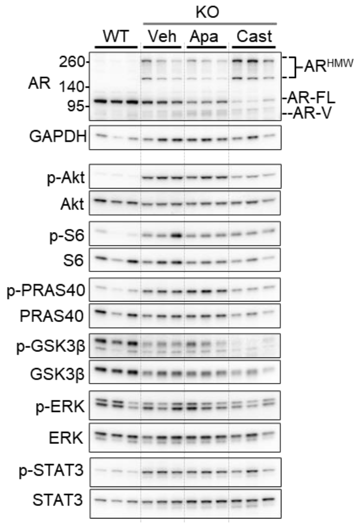

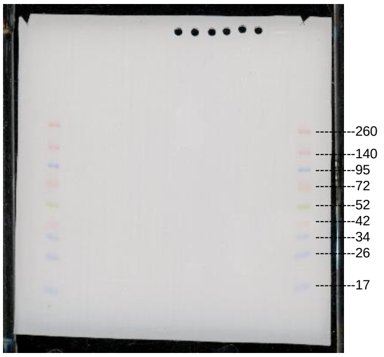

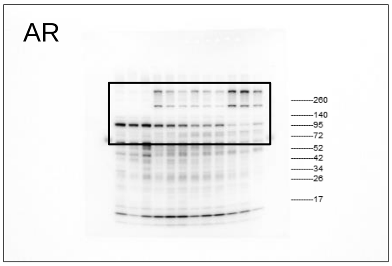

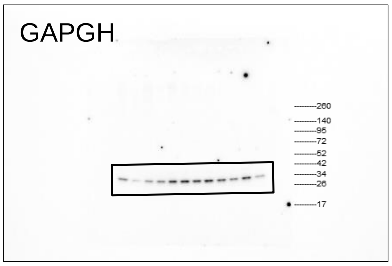

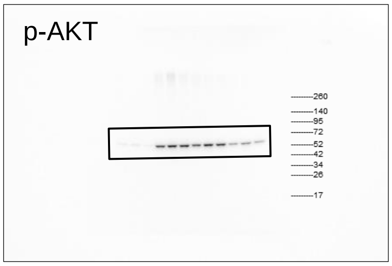

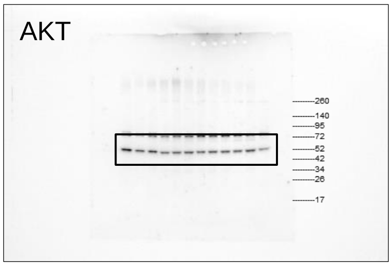

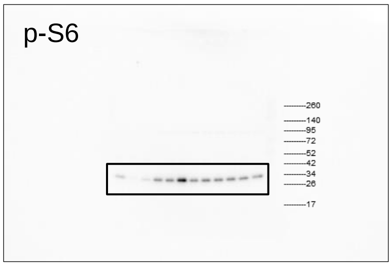

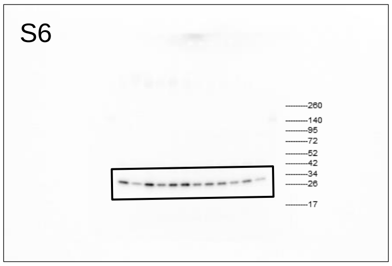

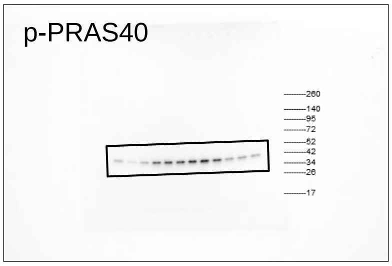

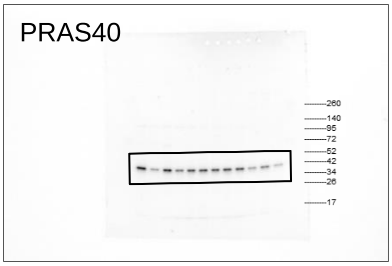

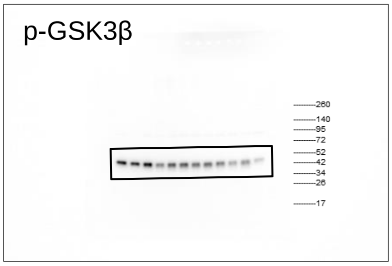

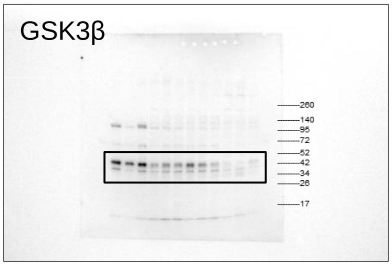

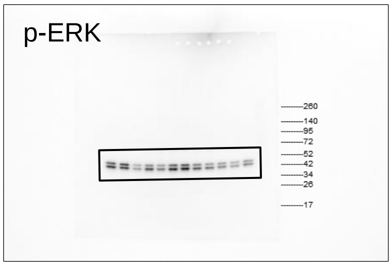

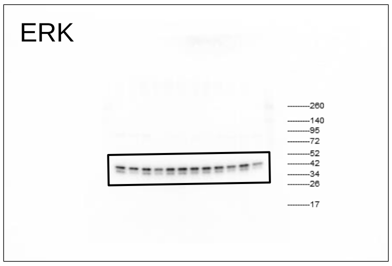

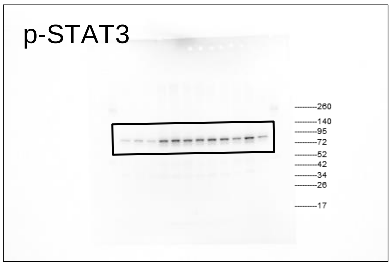

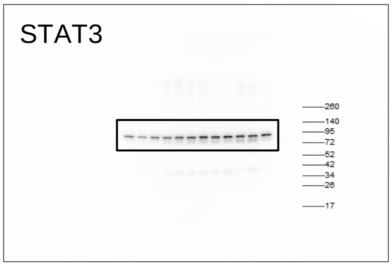

De Velasco et al.

Full unedited membranes for Figure 4B.

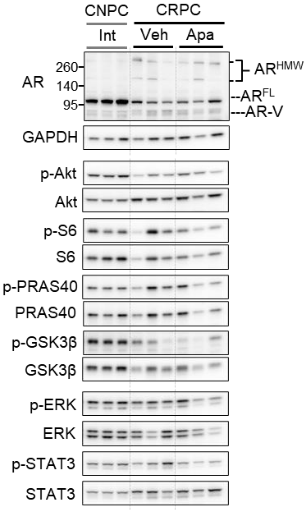

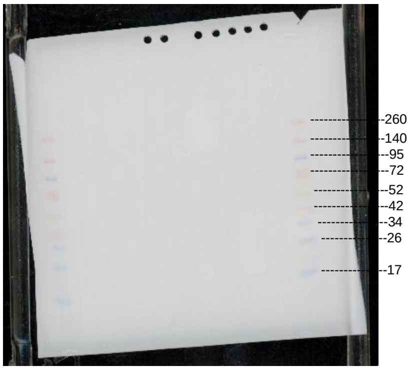

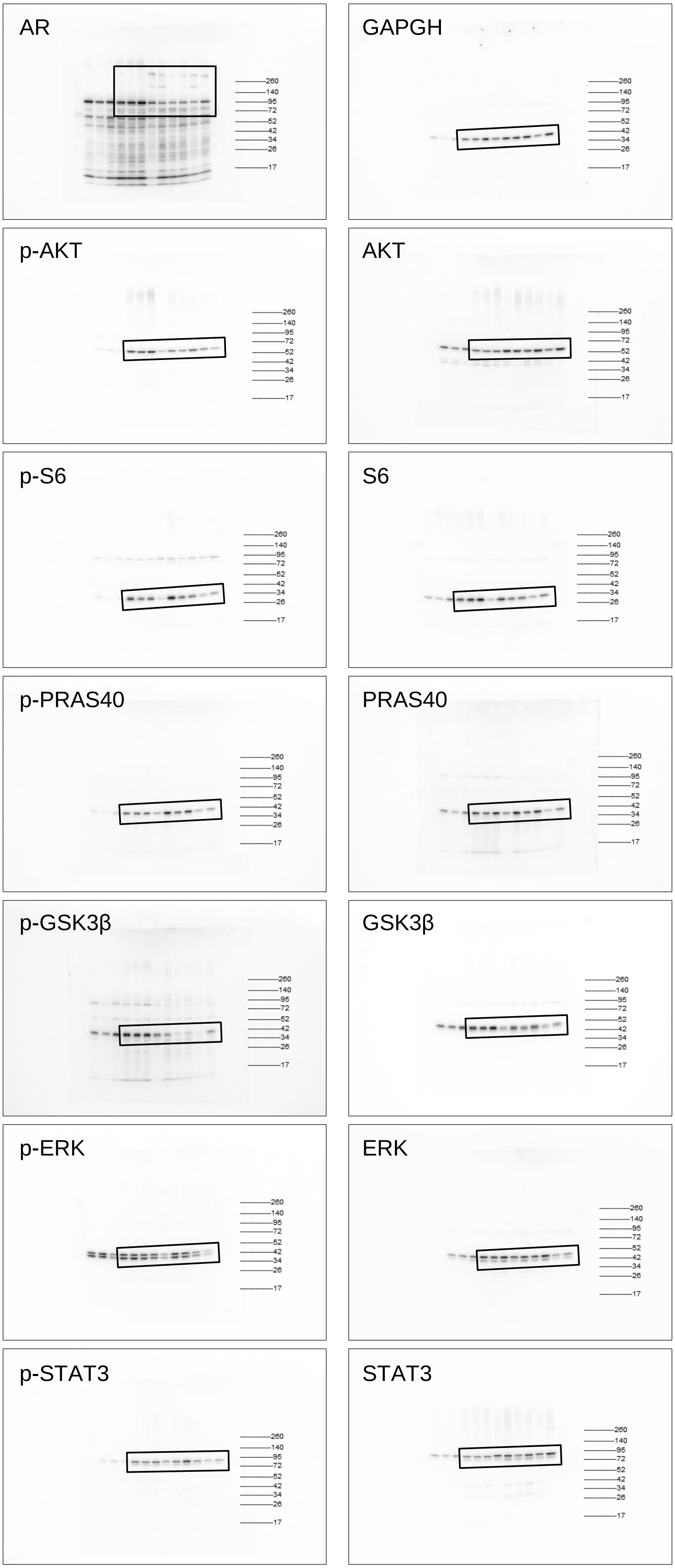

De Velasco et al.

Full unedited membranes for Figure 7A (CNPC).

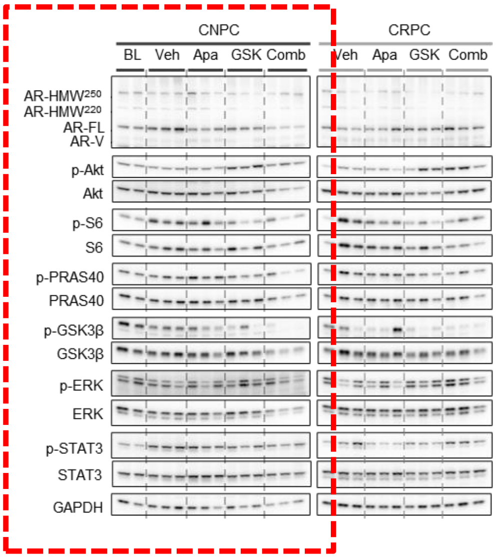

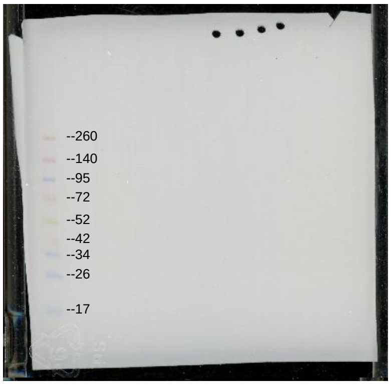

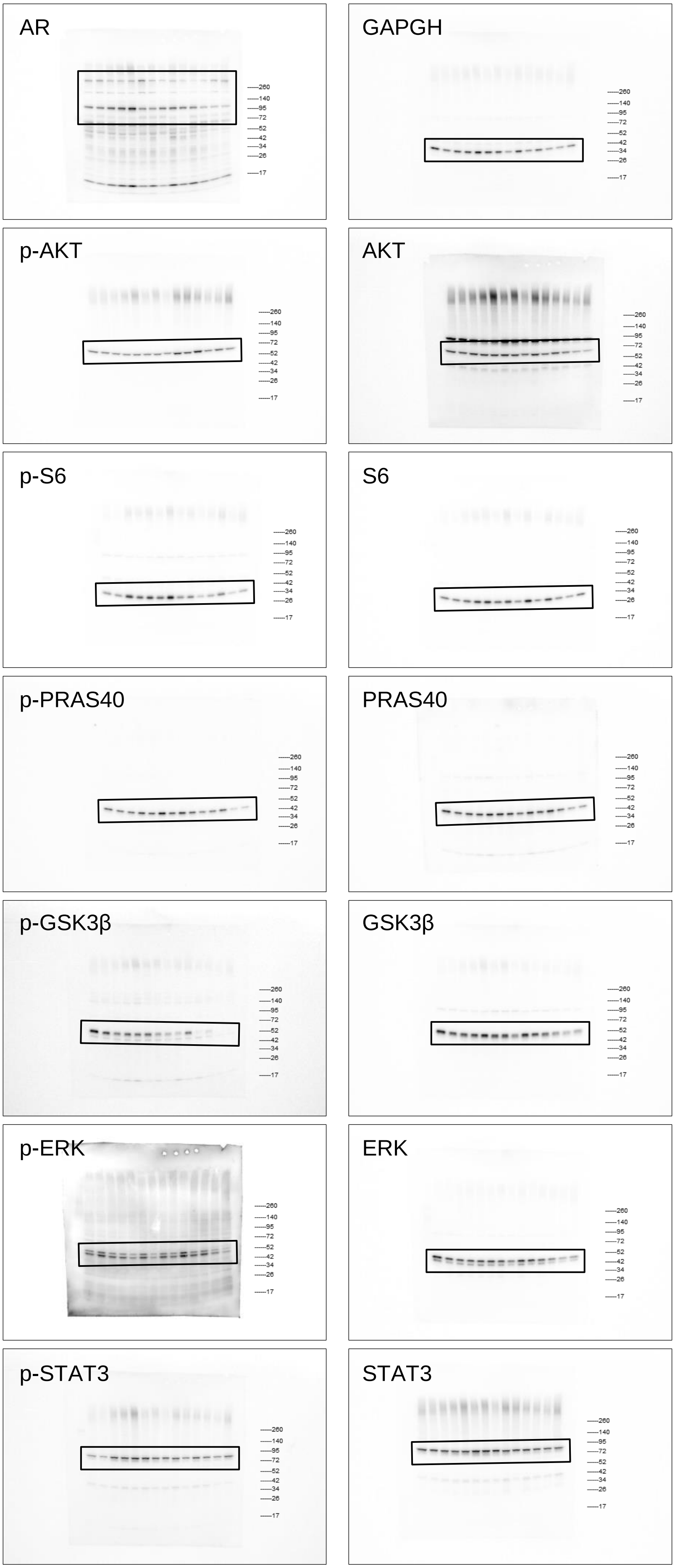

De Velasco et al.

Full unedited membranes for Figure 7A (CRPC).

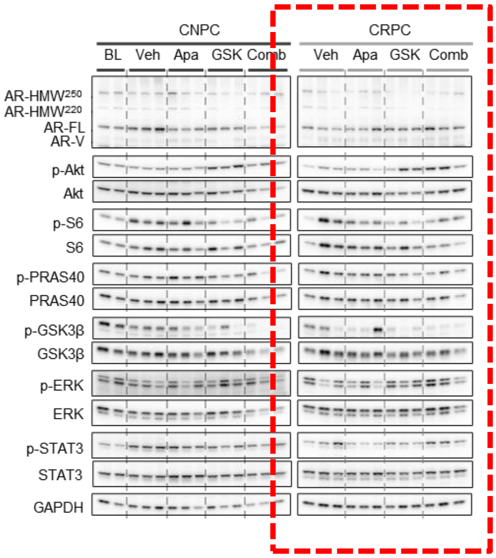

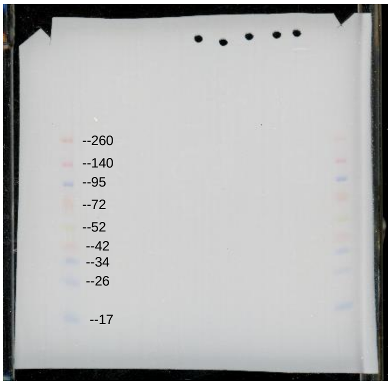

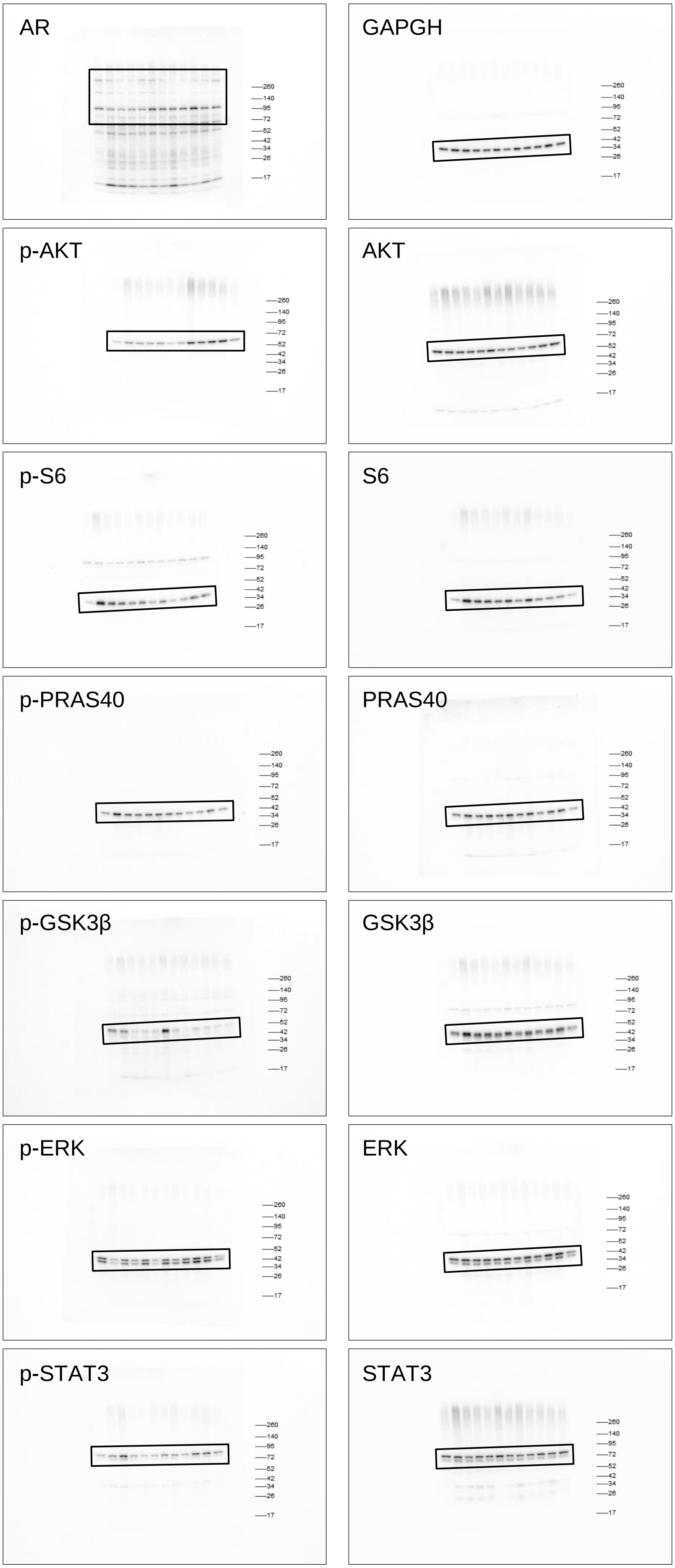

Supplement: Supplementary file 1 [file cancers-13-03975-s001.zip › cancers-1293015_ucroppedWB_forXML.pdf]
